# Vasoactive Intestinal Polypeptide-Immunoreactive Interneurons within Circuits of the Mouse Basolateral Amygdala

**DOI:** 10.1523/JNEUROSCI.2063-17.2018

**Published:** 2018-08-01

**Authors:** Thomas Rhomberg, Laura Rovira-Esteban, Attila Vikór, Enrica Paradiso, Christian Kremser, Petra Nagy-Pál, Orsolya I. Papp, Ramon Tasan, Ferenc Erdélyi, Gábor Szabó, Francesco Ferraguti, Norbert Hájos

**Affiliations:** ^1^Department of Pharmacology,; ^2^Department of Radiology, Medical University of Innsbruck, 6020 Innsbruck, Austria,; ^3^Ledület Laboratory of Network Neurophysiology, Institute of Experimental Medicine, and; ^4^Medical Gene Technology Unit, Institute of Experimental Medicine, Hungarian Academy of Sciences, 1083 Budapest, Hungary

**Keywords:** basolateral amygdala, connectivity, disinhibition, GABA, interneurons, synapse

## Abstract

In cortical structures, principal cell activity is tightly regulated by different GABAergic interneurons (INs). Among these INs are vasoactive intestinal polypeptide-expressing (VIP+) INs, which innervate preferentially other INs, providing a structural basis for temporal disinhibition of principal cells. However, relatively little is known about VIP+ INs in the amygdaloid basolateral complex (BLA). In this study, we report that VIP+ INs have a variable density in the distinct subdivisions of the mouse BLA. Based on different anatomical, neurochemical, and electrophysiological criteria, VIP+ INs could be identified as IN-selective INs (IS-INs) and basket cells expressing CB1 cannabinoid receptors. Whole-cell recordings of VIP+ IS-INs revealed three different spiking patterns, none of which was associated with the expression of calretinin. Genetic targeting combined with optogenetics and *in vitro* recordings enabled us to identify several types of BLA INs innervated by VIP+ INs, including other IS-INs, basket and neurogliaform cells. Moreover, light stimulation of VIP+ basket cell axon terminals, characterized by CB1 sensitivity, evoked IPSPs in ∼20% of principal neurons. Finally, we show that VIP+ INs receive a dense innervation from both GABAergic inputs (although only 10% from other VIP+ INs) and distinct glutamatergic inputs, identified by their expression of different vesicular glutamate transporters.

In conclusion, our study provides a wide-range analysis of single-cell properties of VIP+ INs in the mouse BLA and of their intrinsic and extrinsic connectivity. Our results reinforce the evidence that VIP+ INs are structurally and functionally heterogeneous and that this heterogeneity could mediate different roles in amygdala-dependent functions.

**SIGNIFICANCE STATEMENT** We provide the first comprehensive analysis of the distribution of vasoactive intestinal polypeptide-expressing (VIP+) interneurons (INs) across the entire mouse amygdaloid basolateral complex (BLA), as well as of their morphological and physiological properties. VIP+ INs in the neocortex preferentially target other INs to form a disinhibitory network that facilitates principal cell firing. Our study is the first to demonstrate the presence of such a disinhibitory circuitry in the BLA. We observed structural and functional heterogeneity of these INs and characterized their input/output connectivity. We also identified several types of BLA INs that, when inhibited, may provide a temporal window for principal cell firing and facilitate associative plasticity, e.g., in fear learning.

## Introduction

The basolateral amygdaloid complex (BLA), an extension of the cortex deep in the temporal lobe, has been shown to contribute to a bewildering variety of behavioral functions. These include regulation of goal-directed and social behaviors as well as the formation and storage of affective memories (Phelps and LeDoux, 2005). In this regard, the BLA has been found to be critical in the formation of long-lasting fear memories (LeDoux, 2000; Maren, 2001; Tovote et al., 2015). The BLA consists of the lateral (LA), basal (BA), and accessory basal nuclei, and is composed of ∼85% pyramidal-like principal neurons and ∼15% GABAergic interneurons (INs; McDonald, 1992). The LA and BA nuclei can be further divided into nuclear subdivisions based on distinct neurochemical and cytoarchitectonic features (Martínez-García et al., 2012), but also internuclear and intranuclear connections (Pitkänen and Amaral, 1991; Pitkänen et al., 1997).

Although the BLA has been intensely studied, its intrinsic neural circuits remain poorly understood. One characteristic of BLA INs that has not been fully explored is its diversity. BLA INs vary significantly in their firing, molecular, and morphological properties, including axonal projections (Spampanato et al., 2011; Capogna, 2014; Vereczki et al., 2016; Andrási et al., 2017). Similar to their neocortical and hippocampal counterparts, BLA IN subtypes may serve profoundly different roles in the spatiotemporal control of local network activity associated with different brain states and behavioral contexts (Tremblay et al., 2016).

Most BLA INs are positive for one of the calcium binding proteins, either calbindin or calretinin (CR) (McDonald and Mascagni, 2001; Spampanato et al., 2011). In rats, CR+ INs also express to a certain extent the neuropeptides vasoactive intestinal polypeptide (VIP) and cholecystokinin (CCK; McDonald and Mascagni, 2002; Mascagni and McDonald, 2003) with some overlap between these two subgroups.

In terms of cortical circuitry, recent evidence has suggested that VIP-immunopositive (VIP+) INs have a special role in controlling local microcircuits by preferentially targeting other INs (Acsády et al., 1996; Hajos et al., 1996; Somogyi et al., 2003; Chamberland and Topolnik, 2012; Pfeffer et al., 2013) and thereby effectively disinhibiting projection neurons (Pi et al., 2013). Disinhibition is emerging as a general mechanism not limited to the neocortex (Letzkus et al., 2015). During fear learning, principal neurons in the BLA become disinhibited along their entire somatodendritic tree (Wolff et al., 2014). This disinhibition provides a temporally precise window for enhanced activation, e.g., by concomitantly presented stimuli. VIP+ INs in the neocortex and hippocampus as a population can mediate principal neuron disinhibition. Upon a closer look, these VIP+ INs appear to fall into several distinct types (Acsády et al., 1996; Hajos et al., 1996; Prönneke et al., 2015; He et al., 2016; Tasic et al., 2016) with potentially different roles in circuit function (Fishell and Rudy, 2011). For instance, a portion of hippocampal CCK-positive (CCK+) basket cells coexpress VIP (Acsády et al., 1996; Hajos et al., 1996; Somogyi et al., 2003; Tyan et al., 2014). In the rat BLA, CCK+ basket cells, identified by their expression of cannabinoid (CB) 1 receptors, also coexpress VIP (Katona et al., 2001; Mascagni and McDonald, 2003).

Despite the critical role of VIP+ INs in regulating circuit operation, there have been no studies directly examining VIP+ INs in the mouse BLA so far. The present study was undertaken to provide basic information about the distribution and connectivity of VIP+ INs in the mouse BLA as well as some of their key neurochemical and electrophysiological properties. Because the afferent systems that can recruit VIP+ INs are crucial determinants of the time course and specificity of the ensuing disinhibition, we have also investigated the inhibitory and excitatory inputs to these INs.

## Materials and Methods

### 

#### 

##### Animals.

All procedures involving animals were performed according to methods approved by the Austrian Animal Experimentation Ethics Board (Bundesministerium für Wissenschaft und Verkehr, Kommission für Tierversuchsangelegenheiten) as well as by Hungarian legislation (1998. XXVIII. section 243/1998, renewed in 40/2013.) and institutional guidelines. All procedures complied with the European convention for the protection of vertebrate animals used for experimental and other scientific purposes (ETS number 123). Every effort was taken to minimize animal suffering and the number of animals used. For this study, the following mouse lines were used: C57BL/6J (Charles River), VIPtm1(cre)Zjh (VIP-IRES-cre; RRID:MGI_4436915), Slc32a1tm2(cre)Lowl (VGAT-IRES-cre; RRID:IMSR_JAX:028862), B6.FVB-Tg(Npy-hrGFP)1Lowl/J [neuropeptide Y (NPY)-GFP; RRID:IMSR_JAX:006417], Ai14-cre reporter [Gt(ROSA)26Sor_tm14(CAG/LSL_tdTomato)Hze; RRID:IMSR_JAX:007914], Ai32 [Gt(ROSA)26Sor/CAG/LSL_chr2(H134R)/eyfp (Tm); RRID:IMSR_JAX:024109; Jackson Laboratory], GAD65-EGFP, and BAC-CCK-DsRed. Details on VIP-IRES-cre (Taniguchi et al., 2011), VGAT-IRES-cre (Vong et al., 2011), NPY-GFP (Wood et al., 2016), Ai14 and Ai32 (Madisen et al., 2010), GAD65-EGFP (López-Bendito et al., 2004), and BAC-CCK-DsRed (Máté et al., 2013) mouse lines have been published previously.

##### Surgical procedures and viral vectors.

Anesthesia was induced with 120 mg/kg ketamine and maintained with 1–2% vaporized sevoflurane. Mice were secured in a stereotaxic frame and injections were aimed at the following coordinates: 1.3 mm posterior to bregma; 3.4 mm lateral to the midline; and 4.5 mm deep from the cortical surface. A total of 500 nl of AAV2/6-CBA-FLEX-GFP (Murray et al., 2011; flow rate: 50 nl/min) was unilaterally injected into the BLA of 12-week-old homozygote VIP-IRES-cre mice. In addition, a total of 300 nl of AAV2/5-EF1-DIO-hChR2(H143R)-mCherry (University of North Carolina Vector Core Facility) was bilaterally injected into the BLA of 5–6-week old offspring produced by crossing VIP-IRES-cre and GAD65-EGFP mice. The cannula was slowly withdrawn 5 min after injection. GFP and ChR2 expression was allowed for 2 and 4–5 weeks, respectively, before the animals were killed.

##### Tissue preparation for immunocytochemistry.

After being anesthetized with thiopental (120 mg/kg; Sandoz), adult male mice were transcardially perfused with 0.9% NaCl followed by an ice-chilled fixative solution. For fluorescence microscopy, a fixative solution composed of 2% paraformaldehyde (PFA) and 15% saturated picric acid in 0.1 m phosphate buffer (PB) was used. On the other hand, for horseradish peroxidase (HRP) reactions, the fixative solution contained 4% PFA and 15% saturated picric acid in 0.1 m PB, pH 7.4. The same fixative was also used for pre-embedding electron microscopy (EM) with the addition of 0.05% glutaraldehyde. Brains were immediately removed from the skull, washed in 0.1 m PB, and sliced coronally in 50-μm-thick sections for light microscopy or in 70-μm-thick sections for EM on a Leica VT1000S vibratome (Leica Microsystems). Sections were stored in 0.1 m PB containing 0.05% sodium azide at 6°C until further use. Brains processed for EM were first cut in 6–8-mm-thick blocks, cryoprotected in a solution of 20% sucrose in 0.1 m PB, and freeze-thawed once before sectioning.

##### Antibody characterization.

All primary and secondary antibodies used in this study are listed in Tables 1 and 2. The rabbit polyclonal VIP antibody (ImmunoStar, catalog #20077, lot #1339001) was raised against a synthetic VIP peptide. The specificity of the antiserum was confirmed by soluble preadsorption test with VIP at a concentration of 10^−5^ m, which abolished VIP immunolabeling (Sloviter and Nilaver, 1987; manufacturer's information). The other rabbit polyclonal VIP antibody used in this study has been characterized previously (Köves et al., 1990).

**Table 1.** Primary antibodies used in this study

Antigen	Source	Catalog No.	Lot No.	Host	Dilution	RRID
Bassoon	Abcam	SAP7F407	GR-47339-2	Mouse	1:3000	AB_1860018

		6B3	010399	Mouse	1:20,000	

CR	Swant	7698	21498	Rabbit	1:3000	AB_10000320

					1:1000	

CR	Synaptic Systems	214 104	214104/3	Guinea pig	1:5000 (*in vitro*)	AB_10635160

CaMKII	Abcam	ab52476	—	Rabbit	1:250	AB_868641

	Cayman Chemicals	10006590	04574771	Rabbit	1:1000	AB_409026

CB1 cannabinoid receptor	Frontier Institute	CB1-Go-Af450	—	Goat	1:1000	AB_2571530

CCK	Frontier Institute	CCK8-MO-167-1	167	Mouse	1:1000	AB_2572276

Pro-CCK	Frontier Institute	CCK-pro-Rb-Af350	—	Rabbit	1:1000	AB_2571674

GFP	Molecular Probes/Invitrogen	A11122	1356608	Rabbit	1:1.000	AB_221569

NPY	Synaptic Systems	394 004	394004/1-1	Guinea pig	1:1000	AB_2721083

NOS, brain (NOS-B1)	Sigma-Aldrich	N2280	082M4835	Mouse	1:1000	AB_260754

Parvalbumin	Synaptic System	195 004	195004/9	Guinea pig	1:1000	AB_2156476

RFP	Chromotek	5F8	20904002AB	Rat	1:1000	AB_2336064

Somatostatin	Millipore	MAB354	2984147	rat	1:500	AB_2255365

	ImmunoStar			Rabbit	1:4.000	

VIP	Donated by T. Görcs	20077	1339001	Rabbit	1:20,000	AB_572270

VGAT	Synaptic Systems	131004	—	Guinea pig	1:1000	AB_887873

			23050127			

VGlutT1	Chemicon/Millipore	AB5905	2484243	Guinea pig	1:1000 1:3000	AB_2301751

	Chemicon	AB5907			1:5000	AB_2301731

VGlutT2	Synaptic Systems	135404	23041014	Guinea pig	1:3000	AB_2301731

Voltage-gated potassium channel type 2.1 (Kv2.1)	Neuromab	75-014	449-3AK-78D	Mouse	1:1000	AB_10672253

**Table 2.** Secondary antibodies used in this study

Antibody	Source	Catalog No.	Lot No.	Host	Dilution	RRID
Alexa Fluor 488 anti-guinea pig	Life Technologies	A11073	1637243	Goat	1:1000	AB_142018

Alexa Fluor 488 anti-guinea pig	Jackson ImmunoResearch	706-545-148	118980	Donkey	1:500	AB_2340472

	Jackson ImmunoResearch	715-545-151	—	Donkey	1:500	AB_2341099

Alexa Fluor 488 anti-mouse	Molecular Probes	A21202	913921	Donkey	1:400	AB_141607

Alexa Fluor 488 anti-rabbit	Jackson ImmunoResearch	711-545-152	—	Donkey	1:500	AB_2313584

Biotinylated anti-rabbit IgG	Vector Laboratories	BA-1000	ZA0324	Goat	1:100 EM	AB_2313606

Biotinylated anti-rabbit IgG	Vector Laboratories	711-065-152	66789	Donkey	1:500	AB_2340593

Cy3-conjugated anti-mouse IgG	Jackson ImmunoResearch	715-165-150	67763	Donkey	1:500	AB_2340813

Cy3-conjugated anti-rabbit IgG	Jackson ImmunoResearch	711-166-152	111785	Donkey	1:500	AB_2313568

Cy3-conjugated anti-rat IgG	Jackson ImmunoResearch	712-165-153	113375	Donkey	1:500	AB_2340667

Cy5-conjugated anti- guinea pig	Jackson ImmunoResearch	706-175-148	113929	Donkey	1:500	AB_2340462

Cy5-conjugated anti-rabbit IgG	Jackson ImmunoResearch	711-175-152	108263	Donkey	1:500	AB_2340607

Alexa Fluor 647 anti-rabbit IgG	Jackson ImmunoResearch	711-605-152	99912	Donkey	1:500	AB_2492288

DyL405 anti-chicken	Jackson ImmunoResearch	703-475-155	128385	Donkey	1:500	AB_2340373

DyL405 anti-goat	Jackson ImmunoResearch	705-475-003	112415	Donkey	1:500	AB_2340426

DyL405 anti-guinea pig	Jackson ImmunoResearch	706-475-148	129848	Donkey	1:500	AB_2340470

Gold-conjugated anti-guinea pig IgG	Nanoprobes	2055	15C589	Goat	1:100	—

The mouse monoclonal CCK-8 antibody (Frontier Institute, catalog #CCK8-MO-167-1) was raised against a synthetic CCK-8 peptide, whereas the rabbit polyclonal pro-CCK antibody (Frontier Institute, catalog #CCK-pro-Rb-Af350) was raised against the last 9 aa of the C terminus. These antibodies specifically react with CCK-8 and do not cross-react with gastrin (manufacturer's information).

The guinea pig polyclonal VGluT1 antibody (Millipore, catalog #AB5905, lots #23050127 and #2484243) was raised against the synthetic rat VGluT1 protein. Preadsorption of the VGluT1 antiserum with the immunogen peptide eliminates the immunostaining (manufacturer's information).

Both the guinea pig polyclonal VGluT2 antibody from Millipore Bioscience Research Reagents (catalog #AB5907, lot #23041014) and from Synaptic Systems (catalog #135404) were raised against a synthetic rat VGluT2 protein. Preadsorption of the VGluT2 antiserum with the immunogen peptide eliminates all immunostaining (manufacturer's information).

The rabbit polyclonal GFP antibody (Invitrogen, catalog #A11122, lot #1356608) was raised against GFP directly extracted from *Aequorea victoria*.

Both the mouse monoclonal CR antibody (Swant, catalog #6B3, lot #010399) and the rabbit polyclonal CR antibody (Swant, catalog #7699, lot #18299) were raised against recombinant human CR-22k. The guinea pig CR antibody (Synaptic Systems, catalog #214 104) was raised against mouse CR. The specificity was confirmed in CR knock-out mice (Schiffmann et al., 1999).

The mouse monoclonal neuronal nitric oxide synthase (nNOS) antibody (Sigma-Aldrich, catalog #N2280, lot #082M4835) was raised against a recombinant nNOS fragment (amino acids 1–181) and on Western blot reacted specifically with brain NOS and did not react with NOS derived from macrophages and endothelial cells (manufacturer's technical information).

The rabbit polyclonal CB1 antibody (Cayman Chemicals, catalog #10006590, lot #04574771) was raised against a synthetic peptide from the C-terminal region of the human CB1 receptor (manufacturer's technical information). The antibody labels GABAergic axon terminals (e.g., in the amygdala; Vereczki et al., 2016). The goat polyclonal CB1 antibody (Frontier Institute, catalog #CB1-Go-Af450) was raised against the C-terminal region of the mouse protein CB1 receptor, and the specificity was tested in CB1 knock-out mice (manufacturer's technical information).

The guinea pig polyclonal VGAT antibody (Synaptic Systems, catalog #131004) was raised against the cytoplasmic part of the VGAT protein and the specificity of the antibody has been verified in knock-out mice (manufacturer's technical information).

The mouse monoclonal bassoon antibody (Abcam, catalog #SAP7F407) was raised against a recombinant rat bassoon protein (manufacturer's technical information).

The rat monoclonal RFP antibody (Chromotek, catalog #5F8, lot #20904002AB) recognizes, among other red fluorescent proteins, tdTomato (manufacturer's technical information).

The guinea pig polyclonal NPY antibody (Synaptic Systems, catalog #394 004) was raised against a recombinant mouse NPY (manufacturer's technical information).

The guinea pig polyclonal parvalbumin (PV) antibody (Synaptic Systems, catalog #195 004) was raised against recombinant rat PV (manufacturer's technical information).

The rat monoclonal somatostatin (SOM) antibody (Millipore, catalog #MAB354) was raised against a synthetic SOM peptide (manufacturer's technical information).

The rabbit monoclonal CaMKII antibody (Abcam, catalog #ab52476) was raised against a synthetic peptide of the human CaMKII (manufacturer's technical information).

##### HRP-DAB immunocytochemistry.

Immunocytochemistry experiments were performed according to previously published procedures with minor modifications (Sreepathi and Ferraguti, 2012). Primary antibodies were diluted in a solution containing 2% normal goat serum (NGS), 0.3% Triton X-100 in Tris-buffered saline (TBS), pH 7.4. Free-floating sections were incubated in primary antibodies (Table 1) for 2 d at 6°C and then in secondary antibodies (Table 2) overnight. After extensive washing, sections were incubated in an ABC complex solution (1:100; Vectastain ABC kit, Vector Laboratories) made up in TBS, followed by diaminobenzidine (DAB) as a chromogen (0.5 mg/ml in tris buffer) and 0.003% H_2_O_2_, as the electron donor, for 5 min. The sections were mounted onto gelatin-coated slides, dehydrated in an ascending ethanol series followed by an incubation in butylacetate, and coverslipped with Eukitt (Christine Gröpl, Tulln, Austria).

##### Double immunofluorescence labeling using tyramide signal amplification.

Immunofluorescence experiments were performed according to previously published procedures with minor modifications (Sreepathi and Ferraguti, 2012). Primary antibodies (Table 1) were prepared in 2% NGS and 0.1%Triton X-100 in TBS (TBS-T). After incubation with primary and secondary antibodies (Table 2), the fluorescence signal for VIP was enhanced using a tyramide signal amplification kit (TSA Fluorescein System, PerkinElmer). For the enhancement, sections were first incubated in streptavidin-HRP (diluted 1:500 in 2% NGS in TBS-T) for 30 min at room temperature and then, after two washes in TBS, in a fluorophore tyramide solution for 6 min. Sections were then mounted onto gelatin-coated slides and coverslipped with Vectashield (Vector Laboratories). When mouse monoclonal antibodies were used, sections were pretreated with a Mouse-on-Mouse kit (Vector Laboratories) to reduce endogenous mouse Ig staining.

##### Three-dimensional reconstruction.

Three-dimensional (3D) model reconstructions were made from HRP-DAB stained sections for VIP, covering the full length of the amygdala. Images were acquired through a BX51 microscope (Olympus) equipped with a motorized stage (MAC 6000, MBF Bioscience) and a digital camera (QImaging). Drawings and reconstructions were performed with the Neurolucida software (Version 11, MBF Bioscience, RRID:SCR_001775).

##### Volume correction for tissue shrinkage.

To obtain the tissue shrinkage factor due to tissue fixation, the brain volumes of 13–16-week-old C57BL/6J mice (*n* = 4; 25–30 g) were compared before and after transcardial perfusion with a 3 tesla whole-body MRI device. A resolution of 0.34 × 0.34 × 0.3 mm was obtained with a T2-weighted 3D turbo spin-echo sequence. To guarantee imaging without movement artifacts, the animals were anesthetized with an intraperitoneal injection of ketamine and xylazine (80 mg/kg ketamine and 5 mg/kg xylazine dissolved in a 0.9% sodium chloride solution). Immediately after imaging, the animals were transcardially perfused with 4% paraformaldehyde and 15% picric acid. The brain was removed, placed in PBS-filled falcon tubes, and imaged again. To measure the volume, the image sequences were analyzed with the polygon selection tool of ImageJ (Version 1.48k, RRID:SCR_003070). The volume of the whole brain until the end of the cerebellum was measured and then the volume of the ventricles was subtracted. The volume shrinkage of brain tissue due to fixation was 19.9 ± 3.0%. To determine the shrinkage factor due to the HRP-DAB processing, randomly selected unprocessed sections (*n* = 4) were mounted on an object slide with PBS. Subsequently the section area was measured with the Neurolucida software using a 4× objective. The area of these sections was measured again after HRP-DAB immunolabeling. The area shrinkage factor was 33.4 ± 2.3%.

##### Pre-embedding immuno-EM of AAV2/6-CBA-FLEX-GFP-injected brains.

EM was used to validate light microscopic observations because, while light microscopy gives an estimate of preferred targets, it can be inaccurate in the identification of synaptic contacts (Tamás et al., 1997). Pre-embedding immuno-EM experiments were performed according to previously published procedures with minor modifications (Sreepathi and Ferraguti, 2012). VGluT1 and VGluT2 were visualized by nanogold-silver enhanced reaction. GFP-labeled profiles were revealed by an ABC–HRP reaction. Silver enhancement was always performed first. Fab fragment secondary antibodies coupled to nanogold (1.4 nm) were enhanced with a silver amplification kit (HQ Silver Enhancement Kit, Nanoprobes). Contrast was enhanced using 2% osmium tetroxide v/v (Agar Scientific) in 0.1 m PB for 40 min at room temperature and 1% uranyl acetate w/v (Agar Scientific) in 50% ethanol for 30 min at room temperature. The sections were then dehydrated in increasing gradients of ethanol, immersed in propylene oxide, and embedded in epoxy resin (Durcupan ACM, Sigma-Aldrich) on greased glass slides. Regions of interest were dissected under a stereomicroscope and re-embedded in Durcupan ACM. Ultrathin sections (70 nm) were cut with an ultramicrotome (Ultracut S, Leica Microsystems) and collected on Formvar-coated copper slot grids. The ultrastructural analysis of the specimens was performed using a Philips CM 120 electron microscope equipped with a Morada CCD transmission EM camera (Soft Imaging Systems).

##### Quantification of VIP+ neuron and bouton density.

The density of VIP+ cell bodies and boutons (*n* = 8 amygdalae) was calculated on images of HRP-DAB sections immunolabeled for VIP by using the Neurolucida software. Borders of the LA and BA nuclei were outlined according to the pattern revealed by immunocytochemistry. The nuclear subdivisions were identified with the help of a mouse brain atlas (Franklin and Paxinos, 2007). The total number of VIP+ neurons was determined by counting VIP+ neurons in all sections containing the BLA. To measure the bouton density, we used a 100× objective and a counting frame (50 × 50 μm). Bouton density was counted in every fourth section from three nonoverlapping counting frames per subdivision and per section using the Neurolucida software. To ensure that the neurons and boutons were counted as accurately as possible, the focus was maintained through the whole thickness of each section.

##### Colocalization of VIP with other neuronal markers.

To quantify the colocalization of VIP and CR (*n* = 6 amygdalae), images were taken on an epifluorescence microscope (AxioImager M1, Zeiss) using the Openlab software (Version 5.5.0; RRID:SCR_012158). Images were then imported and analyzed with the Neurolucida software. Each counting field was taken at three different *z* levels and for both channels in every third section. All images were analyzed using nonoverlapping counting frames (150 × 150 μm). For each BLA, a fixed number of counting frames were analyzed: 10 for the dorsolateral subdivision of the lateral nucleus (LAdl), 6 for the ventrolateral subdivision of the lateral nucleus (LAvl), 5 for the ventromedial subdivision of the lateral nucleus (LAvm), 30 for the anterior subdivision of the basal nucleus (BAa), and 14 for the posterior subdivision of the basal nucleus (BAp). The colocalization degree of VIP and CCK (*n* = 6 amygdalae) was obtained by counting all neurons in every third section in the respective subdivision without using counting frames. Similarly, BLA sections at different rostrocaudal levels were tested using a combination of guinea pig anti-PV (Synaptic Systems; 1:3000) and rabbit anti-VIP (Immunostar; 1:1000) and visualized with donkey anti-rabbit Cy3 (1:400) and anti-guinea pig Alexa Fluor 488 (1:1000; Jackson ImmunoResearch).

In a different set of experiments, we calculated the colocalization of CCK and CR in the BLA. Here, CCK-containing and CR-containing neurons were visualized with the rabbit anti-pro-CCK (Frontiers Institute; 1:1000) and guinea pig anti-CR (Synaptic Systems; 1:1000), respectively, using Cy3-conjugated donkey anti-rabbit and Alexa Fluor 488-conjugated donkey anti-guinea pig secondary antibodies. The potential colocalization between VIP and NPY in BLA neurons was analyzed in coronal sections obtained from NPY-GFP mice (Jackson Laboratory) stained with a rabbit anti-VIP (Immunostar; 1:1000) and visualized using a donkey anti-rabbit HRP (Invitrogen; 1:500) and TSA-biotin streptavidin-Dylight 649 (Vector Laboratories).

##### Digital image processing.

Digital images were adjusted using Photoshop CS6 Extended (RRID:SCR_014199) on an Apple computer for contrast, brightness, and tonal values for the entire frame.

##### Estimation of the target distribution of VIP+ varicosities in the amygdala.

To estimate the ratio of the GABAergic and non-GABAergic postsynaptic targets of VIP+ boutons, we used two offspring (P63 and P76) of a VGAT-Cre and Ai14 breeding pair. In these mice, the fluorescent protein tdTomato was selectively expressed in those cells expressing the vesicular GABA transporter (VGAT), i.e., in GABAergic cells. The mice were transcardially perfused with 4% PFA in 0.1 m PB. The brains were resectioned into 50-μm-thick coronal sections, and those containing the amygdala were further processed for immunocytochemistry. Briefly, the amygdala-containing sections were incubated with a solution containing 0.2% Triton X-100 and 10% normal donkey serum (NDS) in 0.1 m PB for 1 h at room temperature. This was followed by a 5-d-long incubation at 4°C in a solution containing 0.2% Triton X-100, 2% NDS, 0.05% sodium azide, and a mixture of primary antibodies: rabbit anti-VIP [courtesy of Professor Tamás Görcs (Köves et al., 1990); 1:20,000], goat anti-CB1 (Frontier Institute; 1:1000), and rat anti-RFP (Chromotek; 1:1000) to enhance the endogenously expressed tdTomato signal. The primary antibodies were visualized using the following secondary antibodies: Alexa Fluor 488-conjugated donkey anti-rabbit (Invitrogen; 1:500), DyL405-conjugated donkey anti-goat (Jackson ImmunoResearch; 1:500), and Cy3-conjugated donkey anti-rat (Jackson ImmunoResearch; 1:500) antibodies. Images from the lateral and basal amygdala were obtained using a Nikon C2 confocal microscope (Plan Apo VC 60× oil objective; numerical aperture, 1.40; *xy*, 0.10 μm/pixel; *z*-step size, 0.25 μm), and analyzed using ImageJ software.

For this analysis, we used the following criteria: (1) VIP+ boutons in close apposition to a tdTomato-expressing profile were considered to establish an apposition onto GABAergic profiles; (2) in those cases in which the tdTomato-containing profile fully overlapped with the VIP+ puncta, the bouton was considered to be a VGAT+ bouton and, therefore, not considered a putative postsynaptic target. The results obtained in the two animals were pooled together, and graphs were made using OriginPro 2015 (RRID:SCR_014212).

##### *In vitro* electrophysiological measurements.

VIP-IRES-cre mice were crossed with Ai14 or Ai32 mice and their litters were used for *in vitro* experiments. Offspring of VIP-IRES-cre::GAD65-EGFP mice that had been injected with the AAV5-DIO-ChR2-mCherry vector were also used for *in vitro* experiments. Mice of both sexes [postnatal days (P) 35–P75] were deeply anesthetized with isoflurane and decapitated. Brains were quickly removed and placed into ice-cold cutting solution containing the following (in mm): 252 sucrose, 2.5 KCl, 26 NaHCO_3_, 1 CaCl_2_, 5 MgCl_2_, 1.25 NaH_2_PO_4_, 10 glucose, bubbled with 95% O_2_/5% CO_2_ (carbogen gas). Coronal slices of 200 μm thickness containing the BLA were prepared with a Leica VT1000S Vibratome, and kept in an interface-type holding chamber containing artificial CSF (ACSF) at 36°C, which gradually cooled down to room temperature. ACSF contained (in mm) 126 NaCl, 2.5 KCl, 1.25 NaH_2_PO_4_, 2 MgCl_2_, 2 CaCl_2_, 26 NaHCO_3_, and 10 glucose, bubbled with carbogen gas.

After ≥1 h of incubation, slices were transferred individually into a submerged-type recording chamber perfused with ACSF at 30 ± 2°C with 2–3 ml/min flow rate. Recordings were performed under visual guidance using differential interference contrast microscopy (Olympus BX61W). tdTomato-containing VIP+ cells in slices obtained from VIP-IREs-cre::Ai14 mice, and GFP-containing GAD65+ cells obtained from VIP-IREs-cre::GAD65-EGFP mice, respectively, were excited by a mercury lamp, and the fluorescent signal was detected by a CCD camera (Hamamatsu Photonics). Patch pipettes were pulled from borosilicate glass capillaries with inner filament (Hilgenberg) using a P-97 Micropipette puller (Sutter Instruments). For whole-cell recordings, pipettes with 0.188 mm wall thickness were used and had a resistance of ∼6–8 MΩ when filled with the intrapipette solution. K-gluconate-based intrapipette solution used in all recordings contained the following (in mm): 110 K-gluconate, 4 NaCl, 2 Mg-ATP, 20 HEPES, 0,1 EGTA, 0.3 GTP (sodium salt), and 10 phosphocreatine adjusted to pH 7.3 using KOH and with an osmolarity of 290 mOsm/L. Biocytin in a concentration of 0.2% was added to the intrapipette solution. Recordings were made with a Multiclamp 700B amplifier (Molecular Devices), low-pass filtered at 3 kHz, digitized at 10 kHz, and recorded with in-house data-acquisition and stimulus software (Stimulog, courtesy of Professor Zoltán Nusser, Institute of Experimental Medicine, Hungarian Academy of Sciences, Budapest, Hungary). Recordings were not corrected for junction potential. To record the firing characteristics, cells were injected with 800-ms-long hyperpolarizing and depolarizing square current pulses with increasing amplitudes from 10 to 600 pA. These voltage responses were analyzed using in-house analysis software SPIN1.0.1 (courtesy of Professor Zoltán Nusser, Institute of Experimental Medicine, Hungarian Academy of Sciences) for the positive steps, and a custom-made program written in Matlab (RRID:SCR_001622) for the negative steps (courtesy of Dr. Szabolcs Káli, Institute of Experimental Medicine, Hungarian Academy of Sciences). Analysis of spiking properties was based on previously published procedures (Antal et al., 2006). Comparison of single-cell properties (including both active and passive membrane characteristics) has been done between groups of neurons categorized based on their neurochemical marker content. These data are reported in Tables 3, 4, and 5. In addition, we rearranged the dataset shown in Table 5 into three groups based on the spiking features of VIP+ INs reported in Figure 5 and regardless of their CR content.

**Table 3.** Single-cell properties of VIP+ IS-INs and CCK+/CB1 + basket cells

	IS-INs	Basket cells	*t*-Student *p* value
Capacitance (pF)	46.92 ± 2.98 (*n* = 30)	88.54 ± 8.16 (*n* = 13)	<0.001*^a^*
Input resistance (MΩ)	350.30 ± 28.26 (*n* = 30)	203.42 ± 27.75 (*n* = 13)	<0.001*^a^*
Membrane time constant (ms)	14.79 ± 0.80 (*n* = 30)	16.12 ± 1.42 (*n* = 13)	0.426
Rheobase first action potential (pA)	43.43 ± 4.80 (*n* = 33)	133.13 ± 16.75 (*n* = 16)	<0.001*^a^*
Action potential threshold (mV)	−37.46 ± 0.67 (*n* = 32)	−38.95 ± 0.89 (*n* = 16)	0.191
Action potential half-width (ms)	0.464 ± 0.032 (*n* = 33)	0.719 ± 0.043 (*n* = 16)	<0.001*^a^*
After-hyperpolarization amplitude (mV)	14.13 ± 0.75 (*n* = 26)	14.71 ± 1.08 (*n* = 16)	0.714

Data are presented as mean ± SEM.

*^a^p* values indicate statistical significance.

**Table 4.** Single-cell properties of CB1+/VIP+ and CB1+/VIP− basket cells

	CB1 + VIP+	CB1 + VIP-	*t*-Student *p* value
Capacitance (pF)	79.57 ± 19.86 (*n* = 4)	92.53 ± 8.48 (*n* = 9)	0.580
Input resistance (MΩ)	225.61 ± 77.75 (*n* = 4)	193.58 ± 24.92 (*n* = 9)	0.717
Membrane time constant (ms)	14.41 ± 3.28 (*n* = 4)	16.88 ± 1.53 (*n* = 9)	0.529
Rheobase first action potential (pA)	132.5 ± 54.4 (*n* = 4)	133.3 ± 15.5 (*n* = 12)	0.989
Action potential threshold (mV)	−40.63 ± 1.12 (*n* = 4)	−38.39 ± 1.11 (*n* = 12)	0.188
Action potential half-width (ms)	0.70 ± 0.08 (*n* = 4)	0.73 ± 0.05 (*n* = 12)	0.806
After-hyperpolarization amplitude (mV)	14.55 ± 1.55 (*n* = 4)	14.77 ± 1.38 (*n* = 12)	0.919

Data are presented as mean ± SEM.

**Table 5.** Single-cell properties of VIP+/CR+ and VIP+/CR− INs

	VIP + CR+	VIP + CR−	*t*-Student *p* value
Capacitance (pF)	53.54 ± 6.75 (*n* = 9)	44.08 ± 3.04 (*n* = 21)	0.227
Input resistance (MΩ)	338.62 ± 45.98 (*n* = 9)	355.30 ± 35.88 (*n* = 21)	0.778
Membrane time constant (ms)	16.62 ± 1.47 (*n* = 9)	14.01 ± 3.28 (*n* = 21)	0.154
Rheobase first action potential (pA)	37.50 ± 7.30 (*n* = 11)	46.52 ± 6.24 (*n* = 22)	0.356
Action potential threshold (mV)	−37.48 ± 1.52 (*n* = 11)	−37.44 ± 0.68 (*n* = 21)	0.982
Action potential half-width (ms)	0.50 ± 0.04 (*n* = 11)	0.45 ± 0.05 (*n* = 22)	0.346
After-hyperpolarization amplitude (mV)	13.03 ± 1.16 (*n* = 11)	15.11 ± 0.95 (*n* = 15)	0.180

Data are presented as mean ± SEM.

For recording IPSPs, postsynaptic principal neurons were held in current-clamp mode at ∼−50 mV [the reversal potential (Erev) of IPSPs using the intrapipette solution containing 4 mm Cl^−^ was found earlier to be ∼−77 mV; Veres et al., 2017]. Bridge balance was adjusted throughout the recordings. Postsynaptic responses in the presence of 10 μm CNQX [an AMPA/kainate (AMPA/KA) type of ionotropic glutamate receptor antagonist] were evoked by three 5-ms-long pluses of blue light at 10 Hz every 30 s using a Polygon 400 (Mightex Systems). After a 10-min-long baseline period, WIN 55,212-2 (1 μm; a cannabinoid receptor agonist) together with CNQX (10 μm) were washed in for 10 min to test the sensitivity of evoked responses for CB1. In four experiments, bath application of WIN 55,212-2 was followed by the wash-in of a mixture containing WIN 55,212-2 and AM 251 (both at 1 μm; the latter is a CB1 antagonist) in the presence of 10 μm CNQX. Recordings were analyzed with EVAN 1.3 (courtesy of Professor István Módy, Department of Neurology and Physiology, UCLA, Los Angeles, California) and Origin 9.2 (OriginLab, RRID:SCR_014212). CNQX disodium salt was added to the superfusate from a 10 mm stock solution dissolved in H_2_O, WIN 55,212-2 was added from a 20 mm stock solution dissolved in 0.1 m HCl, and AM 251 was added from a 10 mm stock solution dissolved in DMSO.

*In vitro* recordings obtained in slices prepared from VIP-IRES-cre::GAD65-EGFP mice injected with AAV-DIO-ChR2-mCherry into the BLA were performed in ACSF and the postsynaptic responses in EGFP+ neurons were evoked by 10-ms-long pulses of blue light applied every 20 s. In these recordings, intrapipette solutions containing 4 mm Cl^−^ (as above) or 60 mm Cl^−^ were used. The latter had a composition of 54 K-gluconate, 4 NaCl, 56 KCl, 2 Mg-ATP, 20 HEPES, 0,1 EGTA, 0.3 GTP (sodium salt), and 10 phosphocreatine in mm adjusted to pH 7.3 using KOH and with an osmolarity of 290 mOsm/L. Biocytin at a concentration of 0.2% was added to the intrapipette solution. To evaluate the magnitude of postsynaptic responses evoked upon light stimulation using both intrapipette solutions, we calculated the conductance of the inhibitory postsynaptic responses (IPSCs) by dividing the peak amplitude of events with the difference between the Erev of IPSCs and the holding potential (i.e., with the driving force). Erev of IPSCs using an intrapipette solution containing 4 mm Cl^−^ was −77 mV (see above), while this value for an intrapipette solution containing 60 mm Cl^−^ was determined experimentally as −31 mV. IPSCs were recorded at a holding potential of −45 and −65 mV having 4 and 60 mm Cl^−^ in the intrapipette solution, respectively. When the light stimulation evoked action current(s) in a postsynaptic neuron [e.g., ChR2 was expressed in an EGFP+ cell (*n* = 7)], data were excluded from further analysis.

##### Immunostaining following *in vitro* recordings.

After recording, slices were fixed overnight by immersion in a solution containing 4% PFA in 0.1 m PB. After fixation, slices were rinsed three times in PB and kept with sodium azide at 4°C for ≤1 week. Biocytin-filled recorded cells were visualized with Cy3-conjugated streptavidin. We found that the intensity of the Cy3-conjugated streptavidin labeling was strong enough to clearly distinguish the processes filled by biocytin from the endogenous tdTomato signal (Vereczki et al., 2016). All cells were imaged using a Nikon C2 confocal microscope (Plan Apo VC 20× objective; numerical aperture, 0.75; *z*-step size, 0.5 μm; *xy*, 0.62 μm/pixel). Those INs (*n* = 12) that had the most axon collaterals in the slice were further used for morphological reconstructions. The dendritic and axonal arbors of the intracellularly filled INs were reconstructed with Neurolucida 10.53 software (RRID:SCR_001775), using confocal stacks acquired from the cell before resectioning. The drawings of each cell were analyzed with Neurolucida Explorer (RRID:SCR_001775), and the values were corrected for shrinkage and flattening of the tissue (correction factor in the *z*-axis: 1.7; no correction in the *x*-axis and *y*-axis). Branched structure analysis was used to study the dendritic/axonal length and number of nodes. Sholl analysis was used to estimate the complexity of the dendritic arbor by determining the number of processes crossing concentric spheres centered on the cell soma with 50 μm increments in their radius. In some cases, slices containing the biocytin-labeled INs were embedded in 1% agar and resectioned into 30-μm-thick sections. The sections were then blocked with 10% NDS (Vector Laboratories) in TBS, pH 7.4. Sections from single slices were divided into two parts that were immunostained differentially. In one case, the following antibodies were used: rabbit anti-CR (Swant; 1:3000), mouse anti-bassoon (Abcam; 1:3000), and guinea pig anti-VGluT1 (Millipore; 1:3000) in TBS with 2% NDS and 0.05% Triton X-100. CR was visualized with Cy5-conjugated donkey anti-rabbit (Jackson ImmunoResearch; 1:500), bassoon was visualized with Alexa Fluor 488-conjugated donkey anti-mouse, and VGluT1 was visualized with DyL405-conjugated donkey anti-guinea pig (Jackson ImmunoResearch; 1:500). In the second case, the following antibodies were used: mouse anti-bassoon (Abcam; 1:3000) and guinea pig anti-VGluT2 (Synaptic Systems; 1:3000). Bassoon was visualized with Alexa Fluor 488-conjugated donkey anti-mouse (Jackson ImmunoResearch; 1:500), and VGluT2 was visualized with DyL405-conjugated donkey anti-guinea pig (Jackson ImmunoResearch; 1:500). Confocal images for investigation of the CR content of VIP+ INs and glutamatergic input estimation (see below) were obtained either with a Nikon C2 confocal microscope (Plan Apo VC 60× objective; numerical aperture, 1.4; *z*-step size, 0.5 μm; *xy*, 0.21 μm/pixel) or with a Nikon A1R confocal microscope (Plan Apo VC 60× objective; numerical aperture, 1.4; *z*-step size, 0.2–0.5 μm; *xy*, 0.06–0.1 μm/pixel).

##### Investigation of the neurochemical marker content of EGFP+ neurons in GAD65-EGFP mice.

Some slices prepared from AAV-injected VIP-IRES-cre::GAD65-EGFP mice were fixed in 4% PFA in 0.1 m PB and after overnight incubation were resectioned at a 40 μm thickness. Different sections were processed for single immunostaining with rabbit anti-pro-CCK (Frontiers Institute; 1:1000), guinea pig anti-NPY (Synaptic Systems; 1:1000), or rabbit anti-CaMKII (Abcam; 1:250). Visualization was performed with Cy5-conjugated secondary antibodies. In addition, double immunostaining was obtained using a mixture of rat anti-SOM (Millipore; 1:500) and guinea pig anti-PV (Synaptic Systems; 1:1000) antibodies or a mixture of rabbit anti-VIP (Immunostar; 1:4000) and guinea pig anti-CR (Synaptic Systems; 1:1000) antibodies. Here, DyL405-conjugated and Cy5-conjugated appropriate secondary antibodies were used to visualize the antigen–antibody complexes. Confocal images were obtained with a Nikon C2 confocal microscope (Plan Apo VC 20× objective; numerical aperture, 0.75; *z*-step size, 2 μm; *xy*, 0.62 μm/pixel) and the analysis was conducted using Neurolucida Explorer (RRID:SCR_001775). Analysis of the colocalization between CCK and EGFP was limited to neurons with large soma, as these are known to be CB1+ basket cells (Szabó et al., 2014) and we did not find overlap between CCK and VIP in these cells in this mouse line. In addition, data for VIP+ and CR+ INs were combined.

##### Estimation of GABAergic inputs received by VIP+ INs.

Two offspring of VIP-IRES-cre mice crossed with Ai14 reporter mice were transcardially perfused with 4% PFA in 0.1 m PB. The brain was removed from the skull and resectioned into 50-μm-thick horizontal slices. To estimate the GABAergic inputs received by tdTomato-expressing VIP+ INs, sections containing the BLA were processed for immunostaining with the following antibodies: guinea pig anti-VGAT (Synaptic Systems; 1:1000), rat anti-RFP (Chromotek; 1:1000), and rabbit anti-CB1 (Cayman Chemicals; 1:1000). VGAT was visualized with Alexa Fluor 488-conjugated donkey anti-guinea pig (Jackson ImmunoResearch; 1:500), tdTomato signal was enhanced by Cy3-conjugated donkey anti-rat (Jackson ImmunoResearch; 1:500), and CB1 was visualized with Cy5-conjugated donkey anti-rabbit antibody (Jackson ImmunoResearch; 1:500). Confocal images were obtained using a Nikon C2 confocal microscope (Plan Apo VC 20× objective; numerical aperture, 0.75; *z*-step size, 1 μm; *xy*, 0.62 μm/pixel). The image analysis was performed using Neurolucida Explorer.

##### Experimental design and statistical analyses.

Statistical tests were performed with the Prism (Version 7.0a for Mac OSX; RRID:SCR_002798) or the Origin software. For two-group comparisons, the Student's *t* test was used for normally distributed datasets and the Mann–Whitney test was used for nonparametric distributions. For multiple comparisons, one-way ANOVA followed by the Bonferroni *post hoc* test was used, whereas for nonparametric distributions, the Kruskal–Wallis test followed by the Dunn's multiple *post hoc* test was applied. Statistical significance was set at *p* < 0.05. Correlations were calculated by using the Pearson correlation test. Distributions (e.g., dendrite diameter targeted by VGluT1+ or VGluT2+ terminals) were analyzed using the two-sample Kolmogorov–Smirnov test.

## Results

### Density and distribution of VIP+ neurons in the mouse BLA

**Figure 1.**
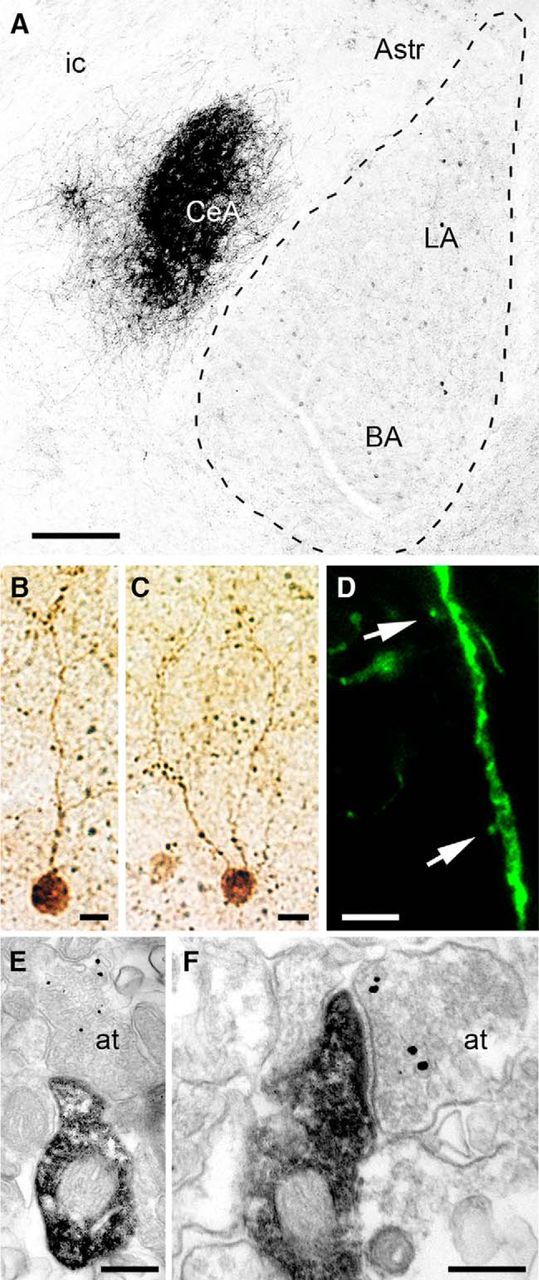
VIP immunoreactivity in the mouse BLA. ***A***, Sparse somata and boutons of VIP+ INs can be observed throughout the BLA, whereas the lateral subdivision of the central amygdala (CeA) shows a dense plexus of VIP-immunolabeled fibers. ***B***, ***C***, Photomicrographs of typical bipolar VIP+ INs as revealed by HRP-DAB immunocytochemistry. ***D***, Photomicrograph of a GFP-labeled dendrite of a VIP+ IN. To visualize the full dendritic domain of VIP+ INs, the viral vector AAV2/6-CBA-FLEX-GFP was injected into the BLA of VIP-IRES-cre mice. The presence in these mice of cre-recombinase only in VIP+ cells enabled the selective expression of the reporter protein GFP. Arrows indicate sporadic spines filled with GFP. ***E***, ***F***, EMs of a stubby spine arising from immunoperoxidase-labeled small VIP+ dendrites. The spine head faces an axon terminal labeled for VGluT1 (gold/silver particles). Astr, Amygdala-striatal transition zone; at, axon terminal; BA, basal nucleus of the amygdala; CeA, central nucleus of the amygdala; ic, internal capsule; LA, lateral nucleus of the amygdala. Scale bars: ***A***, 250 μm; ***B***, ***C***, 20 μm; ***D***, 5 μm; ***E***, 250 nm; ***F***, 200 nm.

In the mouse BLA, the general distribution and morphology of VIP+ INs at the light microscopic level (Fig. 1*A*) was highly similar to those of previous studies in the rat (McDonald, 1985; Muller et al., 2003). VIP immunoreactivity was associated with the somata and primary dendrites of a discrete population of neurons as well as with their axon terminals. A dense plexus of VIP+ axons was also observed in the central lateral amygdaloid nucleus (Fig. 1*A*). Neurons of the BLA containing VIP were mostly bipolar or bitufted having relatively small somata (Fig. 1*B*,*C*). Close immunofluorescence and EM examination of VIP+ neurons showed that although the primary dendrites were mostly aspinous, the additional branches possessed a few stubby spines (Fig. 1*D–F*).

**Figure 2.**
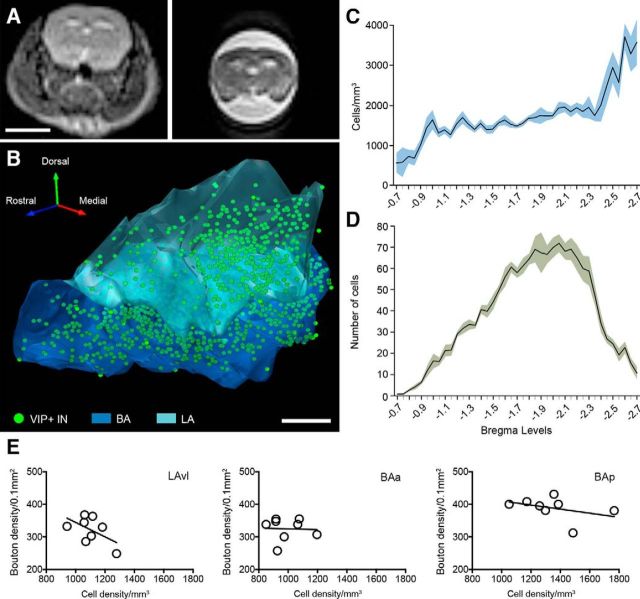
Density of VIP+ INs and boutons in the mouse BLA. ***A***, Magnetic resonance images in the coronal plane of a mouse brain before (left) and after (right) transcardial perfusion obtained using a 3 tesla MRI device. ***B***, 3D reconstruction of the distribution of VIP+ INs (green dots) within the BLA. Note the higher density of VIP+ INs in the more caudal portion of the LA nucleus. ***C***, ***D***, Rostrocaudal density (***C***; cells/mm3) and number (***D***) of VIP+ cells in the mouse BLA. *x*-axis represents all bregma levels analyzed and containing the BLA at 50 μm intervals. *N* = 4 mice. Lineplots depict the mean and the filled area represents SEM. ***E***, Correlation of the density of VIP+ somata and boutons in three different nuclear subdivisions of the BLA. Each dot in the graphs represents the counts from one amygdala in the given subdivision. BA, Basal nucleus of the amygdala; LA, lateral nucleus of the amygdala. Scale bars: ***A***, 5 mm; ***B***, 300 μm.

To get the best approximation of the absolute number and density of VIP+ INs within each subdivision of the LA and BA, we calculated the amygdala shrinkage due to both fixation and immunocytochemical staining procedures. Fixation shrinkage was obtained by comparing the volume of mouse brains before and after transcardial perfusion with a 3 tesla MRI device (Fig. 2*A*). Shrinkage due to processing was obtained by comparing the area of sections before and after immunocytochemical procedures. A volume of 1.497 ± 0.246 mm^3^ (*n* = 16 amygdalae) was obtained for the whole BLA (excluding the basomedial nucleus). The BAa contributed most of the volume (0.665 ± 0.105 mm^3^) followed by the BAp (0.420 ± 0.072 mm^3^), whereas the three LA subdivisions were substantially smaller (LAdl, 0.246 ± 0.036 mm^3^; LAvl, 0.096 ± 0.020 mm^3^; LAvm, 0.071 ± 0.013 mm^3^). To quantify the number of VIP+ neurons, all the sections containing the BLA were immunostained. The BLA was found to contain 1583 ± 89 VIP+ neurons, without a significant difference between the left and right hemispheres (*p* = 0.35, *t* = 1.109, df = 3, *t* test). The density of VIP+ neurons, however, varied significantly among the distinct subdivisions (*p* < 0.0001, *F* = 13.88, df = 4, one-way ANOVA). The LAdl had the lowest density of VIP+ cells (769 ± 131 cells/mm^3^), followed closely by the BAa (990 ± 114 cells/mm^3^). The VIP+ cell density was slightly higher in the LAvm (1094 ± 198 cells/mm^3^) and LAvl (1102 ± 99 cells/mm^3^), whereas the highest density was detected in the BAp (1347 ± 216 cells/mm^3^). We observed a rostrocaudal gradient with fewer VIP+ neurons per mm^3^ rostrally and a higher density caudally (Kruskal–Wallis test, *H* = 147.7, *p* < 0.001; Fig. 2*B*,*C*). However, the absolute number of VIP+ cells was highest in the central sections of the BLA (rostral to caudal: −1.55 to −2.35; Kruskal–Wallis test, *H* = 287.2, *p* < 0.001; Fig. 2*D*). The density of VIP+ axon terminals in the BAa (325 ± 12 terminals/100 μm^2^), BAp (389 ± 12 terminals/100 μm^2^), and LAvl (322 ± 14 terminals/100 μm^2^) was also analyzed and showed a significantly higher density (*p* < 0.01, df = 2, one-way ANOVA with Bonferroni *post hoc* test) in the BAp compared with the other two subdivisions. Surprisingly, when the density of VIP+ somata was related to the density of axon terminals (Fig. 2*E*), no significant correlation could be observed (Pearson correlation coefficient: LAvm, *r* = −0.54, *p* = 0.17; BAa, *r* = −0.04, *p* = 0.92; BAp, *r* = −0.39, *p* = 0.34), which suggests a complex intrinsic and possibly extrinsic innervation pattern of VIP+ axon terminals in the BLA.

### Colocalization between VIP and other neurochemical markers

**Figure 3.**
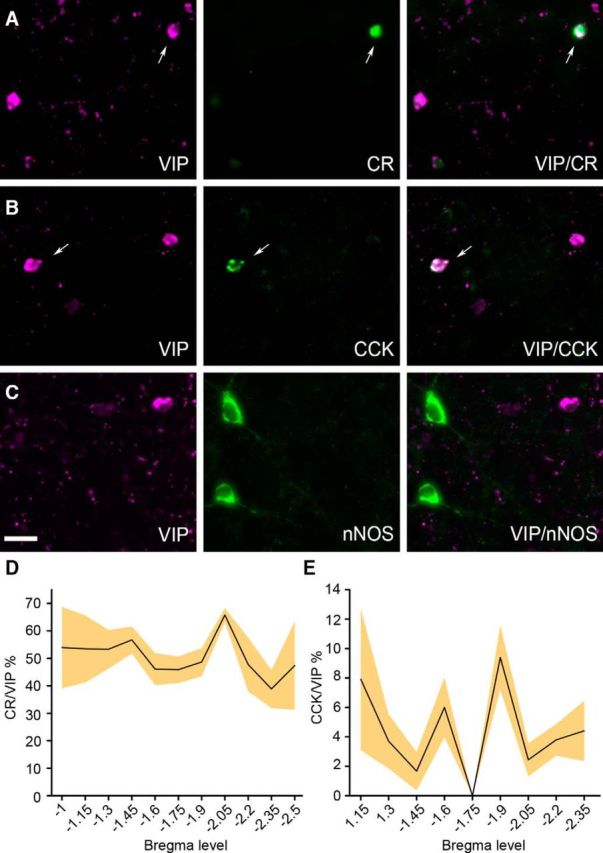
Colocalization of VIP with CR, CCK, or nNOS in the mouse BLA. ***A***, Epifluoresence microscopic images of a neuron colocalizing VIP (magenta) and CR (green) in the BA nucleus. ***B***, Epifluoresence microscopic images of a neuron colocalizing VIP (magenta) and CCK (green) in the BA nucleus. Arrows indicate neurons coexpressing VIP with another marker. ***C***, No colocalization between VIP (magenta) and nNOS (green) was detected in the BLA. ***D***, Percentage of colocalization between VIP and CR along the rostrocaudal axis of the mouse BLA. ***E***, Percentage of colocalization between VIP and CCK along the rostrocaudal axis of the mouse BLA. For all datasets, one in every three sections (50 μm) was analyzed. *N* = 3 mice. Lineplots represent mean, filled areas represent SEM. Scale bar, 50 μm.

In another set of experiments, the colocalization of VIP with other interneuronal markers, such as the calcium-binding protein CR and the neuropeptide CCK, was investigated by double immunofluorescence labeling, as previous studies have shown their coexpression in the rat BLA (McDonald and Pearson, 1989; McDonald, 1994; Mascagni and McDonald, 2003). Our results showed a high degree of colocalization between VIP and CR (Fig. 3*A*), which, however, varied among distinct nuclear subdivisions (*p* = 0.008, df = 4, one-way ANOVA). The highest rate of CR coexpression by VIP+ cells was found in the LAvm (68.11 ± 3.5%, 36 double-labeled cells out of 53) followed by the LAvl (61.04 ± 7.37%, 50 double-labeled cells out of 80) and the LAdl (55.29 ± 7.85%, 73 double-labeled cells out of 133). In the BAa, 47.59 ± 6.97% (200 double-labeled cells out of 411) of the VIP+ cells coexpressed CR, whereas the lowest amount of colocalization was detected in the BAp (41.62 ± 4.77%, 67 double-labeled cells out of 161). The colocalization of VIP with CCK (Fig. 3*B*) was relatively scarce, ranging from ∼12 to ∼3%, and did not statistically differ (*p* = 0.23, df = 4, one-way ANOVA) across the distinct subdivisions of the BLA. The highest rate of CCK coexpression by VIP+ neurons was found in the LAvm (12.01 ± 5.66%, 5 double-labeled cells out of 61), followed by the LAdl (6.66 ± 6.29%, 10 double-labeled cells out of 138), BAp (4.93 ± 2.78%, 11 double-labeled cells out of 223), BAa (3.72 ± 1.4%, 14 double-labeled cells out of 378), and LAvl (2.40 ± 1.70%, 3 double-labeled cells out of 95). Plotting the percentage of VIP+ neurons colocalized with CR or CCK along the rostrocaudal axis revealed a relatively uniform distribution with no significant difference (VIP/CR: one-way ANOVA, *p* > 0.05; VIP/CCK: Kruskall–Wallis ANOVA, *p* > 0.05) detected among the different rostrocaudal bregma levels (Fig. 3*D*,*E*).

Because in the peripheral nervous system VIP+ neurons are also known to coexpress NOS (Chino et al., 2002), we have examined the possible colocalization between VIP and this enzyme. However, no colabeled cells could be detected in the mouse BLA (Fig. 3*C*). No colocalization was also observed between VIP and PV or NPY (data not shown).

### Estimating the ratio of GABAergic postsynaptic targets of VIP+ INs

**Figure 4.**
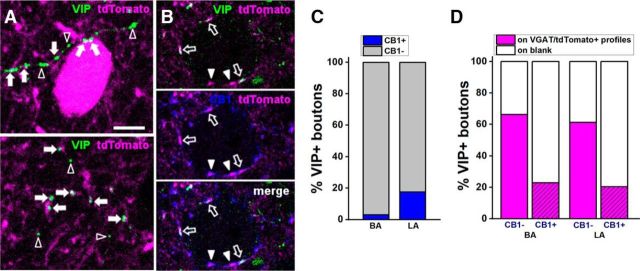
Based on the CB1 content, VIP+ boutons show distinct target preference in the mouse BLA. ***A***, VIP+/CB1− varicosities are often found associated to tdTomato (i.e., GABAergic) profiles in the BLA, revealed in the tissue samples obtained from VGAT-cre::Ai14 mice. Examples of VIP+ boutons in close apposition (white arrows) with a VGAT/tdTomato+ somata (top) and dendrites (top and bottom) in the BA nuclei. Open arrowheads show VIP+ varicosities not associated with VGAT/tdTomato+ profiles. ***B***, Example of VIP+/CB1+ boutons in close apposition (open arrows) with the soma of a non-GABAergic (i.e., principal) neuron in the LA nucleus. White arrowheads point to CB1+/VIP− boutons contacting the same soma. Images in ***A*** and ***B*** have the optical thickness of 0.5 μm. ***C***, Ratio of CB1+/VIP+ and CB1−/VIP+ boutons in the BA and LA nuclei. ***D***, Ratio of CB1− and CB1+/VIP+ boutons that are associated with VGAT/tdTomato+ profiles in the BA and LA nuclei. Blank indicates a profile lacking detectable tdTomato signal. Scale bars, 5 μm.

As VIP+ INs in the hippocampus and dentate gyrus (Acsády et al., 1996; Hajos et al., 1996), as well as in the neocortex (He et al., 2016), form two major groups, i.e., IS-INs and basket cells expressing CCK and CB1 cannabinoid receptors, we first assessed the ratio of VIP+ varicosities that originate from basket cells. To this end, we calculated the percentage of VIP+ boutons that also showed immunoreactivity for CB1. We observed that only a minority of VIP+ axon varicosities may belong to basket cells, as 18% (74 of 419) in the LA and 3% (13 of 424) of VIP+ boutons in the BA, respectively, were found to be immunostained also for CB1 (Fig. 4). These data are overall in agreement with the colocalization results obtained at the soma level (see above) and suggest that the vast majority of VIP+ INs in the BLA are putative IS-INs.

To provide further support for this assumption, we next estimated the ratio of VIP+ boutons immunonegative for CB1 (i.e., that should originate from putative IS-INs), which form close appositions with GABAergic profiles. Through the selective expression of the fluorescent protein tdTomato in GABAergic cells, we could observe that VIP+/CB1− boutons preferentially formed close appositions with tdTomato+ somata and dendrites (Fig. 4). In the LA and BA, 61% (211 of 345) and 66% (272 of 411) of VIP+/CB1− boutons were found to be associated to tdTomato+ profiles, respectively. However, it should be noted that our estimate of the ratio of VIP+/CB1− boutons forming close appositions with GABAergic profiles may be an underestimation, as the tdTomato signal, even after enhancement by immunostaining, might be below the detection threshold in the distal dendrites of GABAergic cells. On the other hand, and in agreement with previous findings (Muller et al., 2003), only a minority of VIP+/CB1+ basket cell boutons contacted tdTomato+ profiles (in the LA, 20%, 13 of 64 boutons; in the BA, 23%, 13 of 57 boutons).

Together these results support the idea that in the BLA, as in other cortical structures, most VIP+ INs preferentially target local GABAergic cells, but only few are basket cells.

### Electrophysiological properties of VIP+ INs

To characterize further BLA VIP+ INs, we studied their active and passive membrane properties by whole-cell recordings in acute slices prepared from VIP-IRES-cre mice crossed with Ai14 reporter mice, in which tdTomato under the control of cre-recombinase was selectively expressed only in VIP+ neurons. All recorded cells, both in the LA nuclei (*n* = 10) and BA nuclei (*n* = 23), were filled with biocytin and visualized, following the recording, for anatomical characterization. VIP+ INs revealed a relatively high input resistance (350.3 ± 28.26 MΩ, *n* = 30) and a small membrane capacitance (46.92 ± 2.98 pF), consistent with their small cell-body size and few proximal dendrites. As shown in Table 3, the recorded VIP+ INs showed very similar passive and active membrane properties. Remarkably, none of the recorded VIP+ INs showed membrane properties compatible with CCK/CB1+ basket cells (Barsy et al., 2017; Rovira-Esteban et al., 2017), suggesting that only IS-INs had been sampled. To address this issue, we recorded CCK+/CB1+ basket cells in slices prepared from BAC-CCK-DsRed transgenic mice and compared their properties with those recorded from VIP+ INs. The two groups of INs indeed differed significantly in several membrane parameters (Table 3), strengthening the conclusion that no CCK+/CB1+/VIP+ basket cell was sampled from slices of VIP-IRES-cre::Ai14 mice. We could further assess the single-cell properties of CCK+/CB1+/VIP+ basket cells, as 4 of 16 neurons recorded in BAC-CCK-DsRed mice showed VIP immunoreactivity in their axonal varicosities. CCK+/CB1+ basket cells containing or lacking VIP immunoreactivity did not differ in any of the parameters measured (Table 4). We did not test the CR content of CCK+/CB1+ basket cells as CCK and CR are basically mutually exclusive in BLA neurons (1 CR+ neuron was found among 162 CCK+ neurons, 1 CCK+ neuron was detected among 174 CR+ cells, *n* = 2 mice).

**Figure 5.**
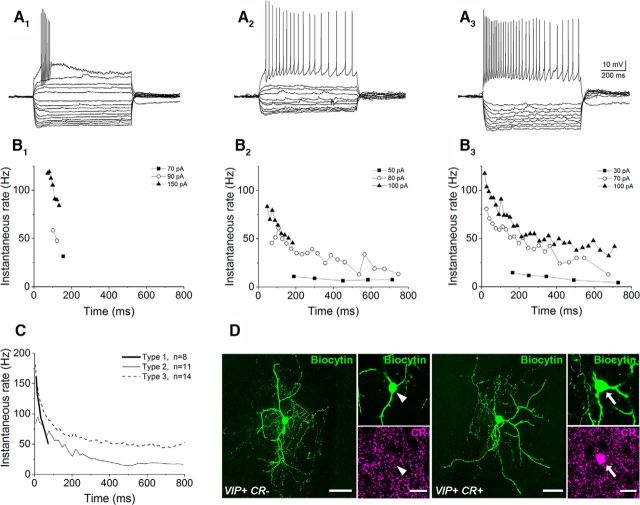
Variability in the firing pattern of VIP+ INs in the mouse BLA. ***A***, Voltage responses to hyperpolarizing and depolarizing step currents of three VIP+ INs. The action potential trains were evoked by injection of a current step with an amplitude of 150 (***A1***), 70 (***A2***), and 100 pA (***A3***), respectively. ***B***, The variability in firing is demonstrated on the time versus instantaneous firing rate plots for the example cells. VIP+ cells discharged only a few action potentials at the beginning of the 800-ms-long current steps (***B1***), spiked throughout the current step at a low rate [in some cases, with spiking restricted to the beginning upon injections of current pulses with large amplitude (≥100 pA); ***B2***], or fired at a high rate (***B3***). ***C***, Summary plot of the firing ability of the cells, when subdivided in three categories, obtained by current injections with amplitude of 70–150 pA. The lines indicate the means. ***D***, Two examples of VIP+ INs that lack or show immunoreactivity for CR in their somata. Scale bars: ***D***, maximal intensity projection image of the example cells, 40 μm; image of the soma, 20 μm.

Upon current pulse injections, VIP+ INs recorded from slices of VIP-IRES-cre::Ai14 mice diverged substantially in their firing characteristics (Fig. 5). Three spiking patterns could be recognized based on the maximum number of spikes emitted upon depolarization and the length of the spike train during the depolarizing current pulses (*p* < 0.001, Kruskal–Wallis ANOVA). Eight cells fired <10 action potentials during the 800-ms-long current pulses (average number of spikes, 6.9 ± 0.8; range, 3–10), a spiking that was restricted to the first 200 ms of the depolarizing steps (Fig. 5*A1*,*B1*,*C*). A second group of cells fired throughout the whole current pulses, but emitted <20 spikes at their maximal firing rate (average number of spikes, 15.4 ± 1.6; range, 7–20; *n* = 11; Fig. 5*A2*,*B2*,*C*). The third cell category fired throughout the depolarizing pulses (average number of spikes, 41.1 ± 3.2; range, 31–63; *n* = 14; Fig. 5*A3*,*B3*,*C*). Statistical comparison of the passive and active membrane properties, however, revealed no difference among these three cell categories (*p* > 0.1, Kruskal–Wallis ANOVA). As nearly 50% of VIP+ INs coexpress CR, we examined the presence of this calcium binding protein in the soma and/or axon varicosities of the recorded and biocytin-filled cells. Of 20 cells tested, 11 were found to be immunopositive for CR (Fig. 5*D*). The presence of CR, however, did not seem to make any distinction among VIP+ cells with different firing patterns (three of six in the short spiking category, three of five among cells with a low spiking rate, and five of nine among cells with a high spiking rate). We also assessed whether the single-cell properties differed between VIP+/CR+ and VIP+/CR− IS-INs. The comparison revealed no significant difference in any of the parameters tested (Table 5). Therefore, VIP+ IS-INs have similar characteristics regardless of their CR content.

**Figure 6.**
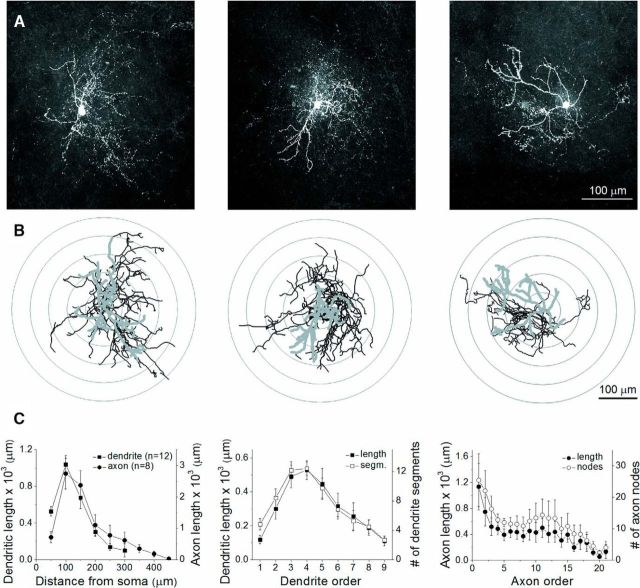
Morphological properties of VIP+ INs. ***A***, Maximum projection intensity images of the three biocytin-filled VIP+ INs, whose firing properties are shown in Figure 5. ***B***, Neurolucida reconstructions of the three INs (dendrites, gray; axon, black). Concentric circles drawn on the reconstructions illustrate the radii used for Sholl analysis. ***C***, Morphological features of dendritic and axonal arbors obtained by analysis of 12 and 8 VIP+ INs, respectively. Each data point represents a mean and SEM.

Furthermore, the visualization of the biocytin content of the recorded cells enabled us to study in more detail the morphological properties of VIP+ INs. Their reconstruction using the Neurolucida software did not reveal any obvious difference among VIP+ cells belonging to the three spiking categories (Fig. 6*A–C*). Overall, we confirmed that VIP+ INs are rather small cells. The total length of the dendrites and the axon collaterals were found to be 2312.5 ± 229.4 μm (*n* = 12) and 7364.5 ± 1607.3 μm (*n* = 8), respectively. The number of the dendrite nodes (27.7 ± 1.7) and axon nodes (94.6 ± 24.4) was also modest. Sholl analysis revealed that the vast majority of dendrites (81.5%) and axons (78.1%) could be found locally near the soma (≤200 μm; Fig. 6*C*). Remarkably, the individual dendritic and axon segments were similar in average length (Fig. 6*C*), in line with observations made for VIP+ INs in other cortical regions (Prönneke et al., 2015; He et al., 2016) and an indication that these INs innervate a rather confined area within the BLA, hence affecting only a few cells nearby.

### Inhibitory inputs of VIP+ INs onto principal neurons are cannabinoid sensitive

**Figure 7.**
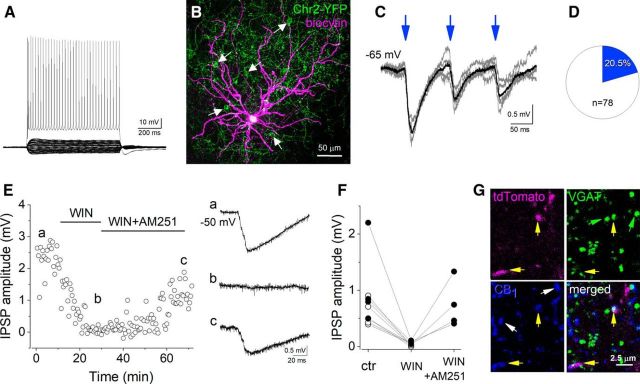
A fraction of principal neurons in the BLA receives cannabinoid-sensitive inhibitory postsynaptic inputs from VIP+ INs. ***A***, ***B***, Voltage responses to hyperpolarizing and depolarizing current steps of a principal neuron (***A***) and its maximum intensity projection image taken after the biocytin visualization (***B***). White arrows indicate the somata of ChR2-YFP-expressing VIP+ INs. ***C***, Large IPSPs at the resting membrane potential upon three 5-ms-long blue-light pulses induced at 10 Hz could be recorded in the principal neuron shown in ***B***. Five consecutive light-evoked events are indicated in gray, while their average is in black. ***D***, In one-fifth of the recorded principal neurons, IPSPs evoked by blue-light stimulation could be identified. ***E***, Bath application of the cannabinoid receptor agonist WIN 55,212-2 (1 μm) eliminated the light-evoked IPSPs in the same principal neuron shown in ***B***, an effect that the coapplication of WIN 55,212-2 together with the CB1 antagonist AM 251 (both applied at a concentration of 1 μm) partially reversed. IPSPs in ***a–c*** are averaged records of five events taken at the labeled time points. ***F***, In all cases (*n* = 9), the light-evoked IPSPs in principal neurons were significantly suppressed by bath application of 1 μm WIN 55,212-2. Coapplication of WIN 55,212-2 and AM 251 (*n* = 4) reversed the WIN 55,212-2-induced suppression of the evoked IPSP amplitude (solid circles). Open circles indicate recordings when only WIN 55,212-2 was bath applied. Each data point is an average of the amplitude of 9–10 consecutive events. ***G***, Single-plane images (with an optical thickness of 0.06 μm) show two tdTomato-labeled varicosities in the BA nuclei that also express VGAT and CB1 (yellow arrows). Green arrows point toward VGAT+ boutons, while white arrows indicate two boutons expressing both CB1 and VGAT, but not tdTomato.

In agreement with previous studies in the hippocampus (Acsády et al., 1996; Hajos et al., 1996; Tyan et al., 2014), amygdala (Muller et al., 2003), and neocortex (Staiger et al., 2004; Pfeffer et al., 2013), our data indicate that a fraction of VIP+ INs belongs to basket cells expressing CCK and CB1. To test whether principal neurons in the amygdala indeed receive functional innervation from CCK+/CB1+/VIP+ basket cells, we performed *in vitro* recordings combined with optogenetics. In acute slices prepared from offspring of VIP-IRES-cre and Ai32 mouse lines, we recorded principal neurons using whole-cell mode (Fig. 7*A–C*). In the presence of an AMPA/KA ionotropic glutamate receptor antagonist, CNQX, we observed IPSPs evoked by whole-field blue-light illumination in ∼20% (Fig. 7*D*) of the recorded cells (*n* = 78; IPSP amplitude, 1.104 ± 0.275 mV, *n* = 16). Bath application of the CB1 agonist WIN 55,212-2 (1 μm) abolished the IPSPs (6.6 ± 1.6% of control amplitude, in control: 0.808 ± 0.185 mV; in WIN: 0.047 ± 0.011 mV, *n* = 9, *p* = 0.002, paired *t* test). In all cases tested, the coapplication of WIN 55,212-2 (1 μm) with the CB1 antagonist AM251 (1 μm) reversed the effects of WIN 55,212-2 on the IPSP amplitudes [control: 1.085 ± 0.38 mV; WIN: 0.072 ± 0.015 mV (7.5 ± 1.4% of control amplitude); WIN+AM251: 0.737 ± 0.212 mV (73.8 ± 11.5% of control amplitude); *n* = 4; control vs WIN effect, *p* = 0.035; WIN vs WIN+AM251, *p* = 0.046; control vs WIN+AM251, *p* = 0.17; Fig. 7*E*,*F*]. To further confirm the GABAergic nature of boutons expressing VIP and CB1, we performed immunostainings against CB1 and VGAT in VIP-IRES-cre::Ai14 mice. Indeed, in all but one tdTomato-expressing VIP+ varicosities immunoreactive for CB1 (*n* = 30), we could detect the presence of VGAT (Fig. 7*G*).

Since most, if not all, principal neurons in the BA receive perisomatic GABAergic inputs from CCK+/CB1+ basket cells (Vereczki et al., 2016), it was surprising to observe that light stimulation of ChR2-expressing VIP+ INs that include basket cells gave rise to evoked responses in only ∼20% of principal neurons. This discrepancy may have two, mutually nonexclusive explanations: first, our recording conditions were not optimal to detect postsynaptic responses in principal neurons originated from CCK+/CB1+/VIP+ basket cells; or second, not all principal neurons receive perisomatic GABAergic input from CCK+/CB1+/VIP+ basket cells. To test the latter scenario, we examined the percentage of BLA principal neurons, identified by their immunoreactivity for Kv2.1 channels (Vereczki et al., 2016), that received close perisomatic appositions by VIP+/CB1+ boutons. CB1+ varicosities contacted the somata of all examined principal neurons, as previously reported (Vereczki et al., 2016), but not all principal cells were targeted by VIP+/CB1+ boutons. Principal cell somata in LA and BA were found to be contacted by ≥1 VIP+/CB1+ varicosity in 59.5 and 74.4% of the cases, respectively. However, there was a marked difference in the ratio of VIP+/CB1+ boutons per cell in these two amygdala nuclei. In LA, nearly half of the CB1+ varicosities (51.8 ± 5.2%, *n* = 240 varicosities in 39 cell somata) that contacted the soma surface of principal neurons showed immunoreactivity for VIP, whereas this ratio was much lower in BA (15.3 ± 1.7%, *n* = 600 varicosities in 38 cell somata).

Together these data indicate that VIP+ basket cells innervate a selected subset of BLA principal neurons, and that they contribute to a different extent to the perisomatic inhibition of principal neurons in the LA and BA.

### VIP+ INs give rise to inhibitory inputs onto different types of BLA INs

**Figure 8.**
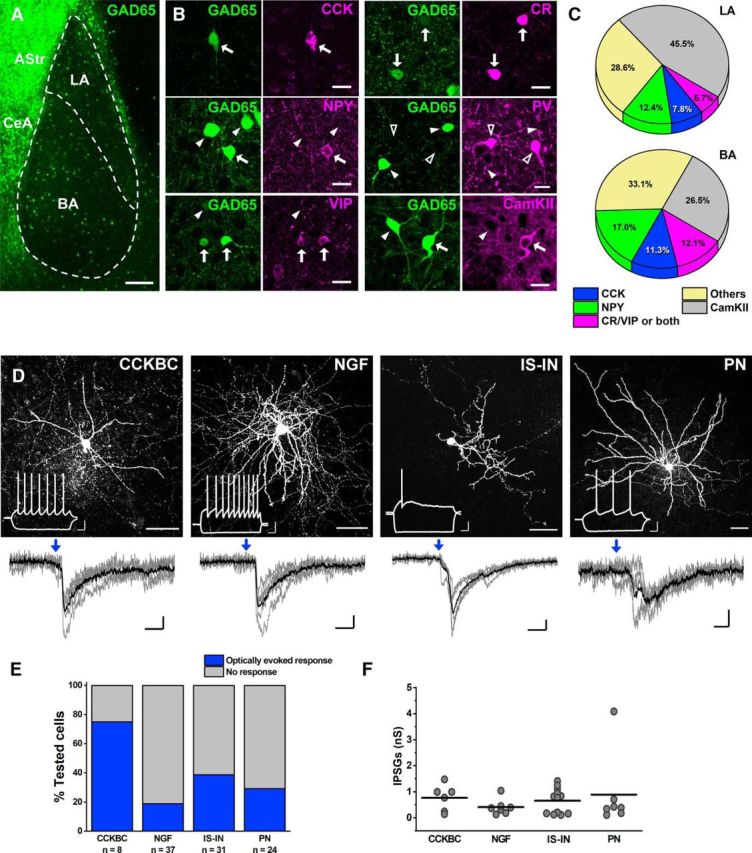
VIP+ INs innervate distinct populations of cells labeled in GAD65-EGFP knock-in mice. ***A***, An image taken from the amygdala region of a GAD65-EGFP mouse. There is a clear difference in the quantity of EGFP-expressing cells between the BLA and striatal-like amygdala regions (CeA, central amygdala; Astr, amygdalostriatal area). ***B***, CCK, NPY, VIP, CR, and CaMKII, but not PV, were found to be expressed in EGFP+ neurons. White arrows show green cells that express the given neurochemical markers, white arrowheads indicate green cells that do not contain the given neurochemical markers, and open arrowheads point to PV-containing cells that were not EGFP+. ***C***, Pie charts show the percentage of EGFP+ neurons containing the given neurochemical markers in both LA and BA nuclei. The “Others” category comprises all green cells that lack the immunoreactivity for the neurochemical markers tested and the three SOM+/EGFP+ neurons. As VIP and CR form partially overlapping groups of INs, they were pooled together. ***D***, Maximum projection intensity images of representative biocytin-filled neurons from four cell types expressing EGFP. Insets show their characteristic responses upon step current injections. CCKBC, CCK+ basket cell; NGF, neurogliaform cell; PN, principal neuron. Below each image, the postsynaptic currents evoked by whole-field blue-light illumination with high chloride intrapipette solution are shown. Blue arrows show the beginning of a 10-ms-long light illumination. Gray traces indicate five consecutive events, while the black traces are their averages. ***E***, Bar graph shows the percentage of different types of EGFP+ neurons in which blue light-evoked postsynaptic events could be detected. ***F***, Plot shows the magnitude of inhibitory postsynaptic responses recorded in different types of EGFP+ neurons (CCK-BC, 0.77 ± 0.21 nS, *n* = 6; NGF, 0.41 ± 0.11 nS, *n* = 7; IS-IN, 0.66 ± 0.14 nS, *n* = 12; PN, 0.89 ± 0.54 nS, *n* = 7). Each dot represents the average peak conductance (IPSG) obtained in individual cells, while horizontal bars indicate the mean. Scale bars: ***A***, 150 μm; ***B***, 20 μm; ***D***, top, 40 μm (inset: *x* = 100 ms, *y* = 20 mV); ***D***, bottom, *x* = 5 ms, *y* = 10 pA).

In a new set of experiments, we examined whether VIP+ INs in the mouse BLA innervate other INs. Previous studies in the neocortex and hippocampus have shown that VIP+ INs innervate GABAergic cells that express PV or SOM (Dávid et al., 2007; Lee et al., 2013; Pfeffer et al., 2013; Pi et al., 2013), a finding that was recently reported also in the BLA (Krabbe et al., 2016). Therefore, we took advantage of a GAD65-EGFP knock-in mouse line, in which INs that contain PV or SOM do not express EGFP (López-Bendito et al., 2004). As the expression of EGFP in this mouse line has never been tested in the BLA, we first examined the neurochemical content of neurons that express this fluorescent protein (Fig. 8*A*). In the BLA, comparable fractions of EGFP+ neurons were found to be immunoreactive for CCK (9.6%, 20 of 208), NPY (13.9%, 28 of 201), and VIP and/or CR (9.2%, 25 of 271, *n* = 2 mice; Fig. 8*B*,*C*). PV+ INs did not contain EGFP (0 of 171 for PV), and only a few EGFP+ cells showed immunoreactivity for SOM (1.7%, 3 of 171 for SOM, *n* = 2 mice; Fig. 8*B*,*C*), in agreement with previous results (López-Bendito et al., 2004). However, we noticed that a considerable fraction of EGFP+ neurons with large somata could not be labeled for any of the markers that have been examined. When these neurons were assayed for the expression of CaMKII, a reliable marker for principal neurons in the BLA (McDonald and Mascagni, 2002), to our surprise, 34.3% of green cells were immunopositive for CaMKII (122 of 356, *n* = 3 mice; Fig. 8*B*,*C*). In addition, a subpopulation of EGFP+ neurons was immunonegative for all neurochemical markers tested. However, these neurons could be false negative, due to low levels of any of the antigens. Thus, in the BLA of GAD65-EGFP mice, in addition to principal neurons, ≥3 largely nonoverlapping populations of INs can be investigated, namely CCK+ basket cells (Rovira-Esteban et al., 2017), NPY-expressing neurogliaform cells (Mańko et al., 2012), and VIP/CR-containing IS-INs (present study).

To test whether these three IN types receive innervation from VIP+ INs, we crossed VIP-cre mice with GAD65-EGFP knock-in mice. Then, into the BLAs of the offspring, we injected a cre-dependent viral construct-transducing ChR2. In agreement with our neurochemical results, the vast majority of recorded neurons could be divided into four groups based on their firing pattern: principal neurons (*n* = 24), CCK+ basket cells (*n* = 8), neurogliaform cells (*n* = 37), and IS-INs (*n* = 31; Fig. 8*D*). The morphological features of recorded neurons, when the visualization of the dendritic and axon arbor was successful (in 58 of 103 cells), confirmed the categorization based on single-cell properties (Fig. 8*D*). In addition, we also recorded from three neurons whose firing pattern was different from that of the other three IN types. Whole-field blue-light stimulation evoked postsynaptic responses in all cell types, but the likelihood, as well as the magnitude, of light-evoked events varied among the cell types (Fig. 8*D–F*). In all three unidentified cells (unINs), light-evoked postsynaptic responses could be also detected (IPSG: 0.12 ± 0.07 nS, *n* = 3). The light-evoked responses were mediated via GABA_A_ receptors, as bath application of gabazine, a selective GABA_A_ receptor antagonist, abolished them (*n* = 9, 4 CCK+ basket cells, 4 IS-INs, and 1 unIN).

Therefore, VIP+ INs in the BLA give rise to GABA_A_ receptor-mediated postsynaptic responses in several INs, including CCK+ basket cells, neurogliaform cells, and other VIP/CR+ IS-INs, although the strength or efficacy of the innervation appeared largely different among postsynaptic neurons.

### GABAergic innervation of VIP+ INs

**Figure 9.**
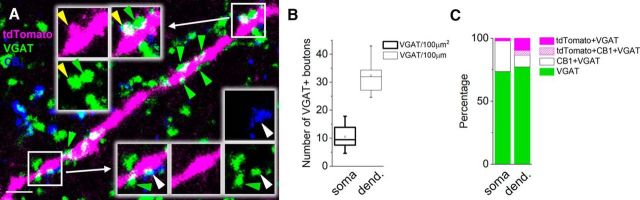
One-quarter of GABAergic inputs onto VIP+ INs originate from other VIP+ and/or CB1+ GABAergic cells. ***A***, Maximum projection intensity image (with an optical thickness of 0.35 μm) of a VIP/tdTomato+ dendrite showing close appositions of several VGAT+ varicosities (green arrowheads), among which VIP/tdTomato/VGAT+ (yellow arrowhead) or CB1/VGAT+ boutons (white arrowhead) could be occasionally observed. Scale bar, 2.5 μm. ***B***, Plot indicates the number of VGAT+ varicosities on the somata and dendrites of VIP+ INs (*n* = 9). The mean (small open square), the median (midline of the box), the interquartile range (box), and the 5/95% values (ends of whisker bars) are shown. ***C***, Percentage of GABAergic boutons expressing VIP/tdTomato and/or CB1 in addition to VGAT. We could detect 219 VGAT+ varicosities on the somata of 9 VIP+ INs, and 223 on their dendrites.

Taking advantage of the recorded slices from VIP-IRES-cre::Ai14 mice, we have estimated the density of GABAergic inputs received by VIP+ INs and the ratio of VIP+ and/or CB1+ boutons among GABAergic varicosities forming close appositions with tdTomato+ profiles (Fig. 9*A*). We found that on the somata of VIP+ INs (*n* = 9), the density of VGAT+ axon terminals was 10.5 ± 1.4/100 μm^2^, while on their dendrites an average of 32.4 ± 2.1 boutons could be detected along a 100-μm-long segment (Fig. 9*B*). Analysis of multicolor stainings revealed that on the somata of VIP+ INs, 2.3% of the total VGAT+ inputs (∼0.25 bouton/100 μm^2^) was also VIP+, and 24.2% (∼2.5 bouton/100 μm^2^) was immunoreactive for CB1 (Fig. 9*C*). No bouton contacting the soma was immunopositive for both tdTomato and CB1. On the dendrites, sampled ≤150 μm from the soma, the ratio of VIP+ GABAergic boutons was higher (9.7%; ∼3 boutons/100 μm), while the proportion of CB1+ GABAergic varicosities was smaller (9.3%, ∼3 boutons/100 μm) compared with that observed on the soma. We also found a small fraction of VGAT+ boutons (3.8%; ∼1 bouton/100 μm) forming appositions to dendrites that were coimmunolabeled for tdTomato and CB1. As each of the nine recorded and labeled VIP+ INs had a small soma, characteristic for IS-INs, these data indicate that <10% of GABAergic inputs onto VIP+ INs preferentially targeting GABAergic cells originate from other VIP+ INs and that ∼15% of inhibitory axon terminals contain CB1.

### Glutamatergic innervation of VIP+INs

**Figure 10.**
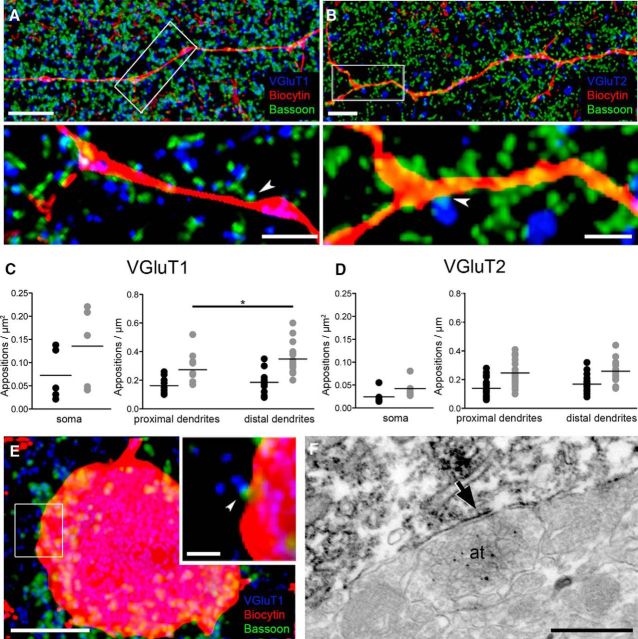
Density of appositions containing VGluT1 or VGluT2 on somata and dendrites of VIP+ INs in the mouse BLA. ***A***, ***B***, Photomicrographs of a distal dendrite labeled for biocytin (red), bassoon (green), and VGluT1 (left) or VGluT2 (right; blue). White arrows indicate terminals with bassoon labeling facing the VIP+ dendrite. ***C***, ***D***, Density of VGluT-containing appositions at the somata, at the proximal dendrites, and at the distal dendrites of VIP+ INs. Black dots correspond to appositions displaying bassoon facing the VIP+ dendrite, whereas gray dots correspond to appositions without bassoon labeling. ***E***, Photomicrograph of the soma of a VIP+ IN receiving a VGluT1+ input (white arrow). ***A***, ***B***, and ***E*** are maximal projections derived from a *z* stack. ***F***, An asymmetric synapse formed between an axon terminal containing VGluT2 and the soma of a VIP+ IN. Scale bars: ***A***, 5 μm (inset, 2 μm); ***B***, 5 μm (inset, 1 μm); ***E***, 5 μm (inset, 1 μm); ***F***, 500 nm. *p < 0.05.

Likewise, to assess the density of glutamatergic inputs onto VIP+ INs, sections from recorded and biocytin-filled cells (*n* = 6) were stained for the VGluT1 or VGluT2 and for the scaffolding protein bassoon to visualize the presynaptic active zone (Fig. 10*A*,*B*). Because of their complementary distribution in the brain, VGluT1 and VGluT2 can be used to discriminate at least in part the origin of glutamatergic inputs (Fremeau et al., 2004). VGluT1 predominates in cortical pyramidal neurons, whereas thalamic and hypothalamic projection neurons mostly have VGluT2 at their efferents (Fremeau et al., 2004). VGluT1+ axon terminals had a density of appositions on VIP+ INs of 7.3 ± 2.5/100 μm^2^ on the somata, an average of 16.0 ± 1.6 μm^2^ on the proximal dendrite, and of 18.0 ± 1.6 μm^2^ on the distal dendrites, as measured along a 100-μm-long segment (Fig. 10*C*). VGluT2+ axon terminals had a similar density of appositions on VIP+ INs with 18.0 ± 1.7 boutons on the proximal and 17.0 ± 1.8 on the distal dendrites per 100 μm (Fig. 10*D*). As we could detect only two VGluT2+ appositions on the somata of VIP+ INs in the analyzed sample, no mean density could be calculated. Based on their morphological and single-cell properties (see above), these biocytin-filled cells likely belong to IS-INs. Hence, our results suggest that VIP+ IS-INs receive inputs from both cortical and subcortical structures.

**Figure 11.**
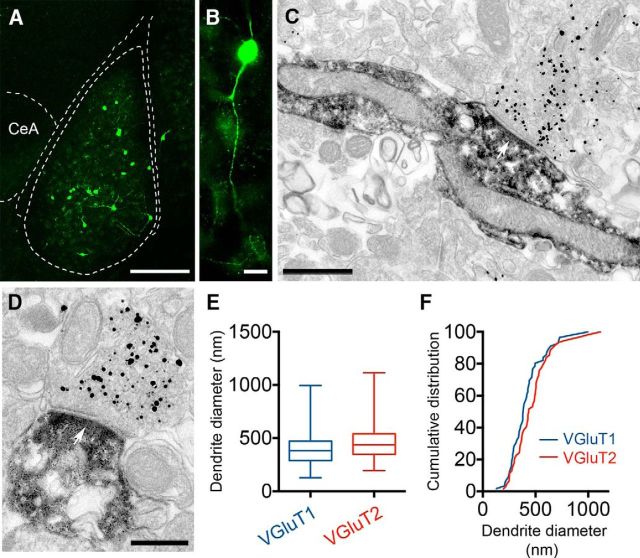
VGluT1+ and VGluT2+ axon terminals form synapses with different caliber dendrites of VIP+ INs of the mouse BLA. ***A***, Injection of an AAV2/6-CBA-FLEX-GFP vector into the BLA of VIP-IRES-Cre mice enabled the expression of the reporter protein GFP in VIP+ INs. ***B***, GFP was detected throughout the somatodendritic domain as well as among axons of VIP+ INs. ***C***, Asymmetric synapse (arrow) formed by an axon terminal containing VGluT1 (gold/silver reaction) with the dendrite of a VIP+ IN (HRP-DAB reaction). ***D***, Asymmetric synapse (arrow) formed by an axon terminal containing VGluT2 (gold/silver reaction) with the dendrite of a VIP+ IN (HRP-DAB reaction). ***E***, Whisker plots indicate the diameter of VIP+ IN dendrites targeted by VGluT1+ (*n* = 56, sampled from 3 animals) or VGluT2+ (*n* = 62, sampled from 3 animals) axon terminals. ***F***, Frequency distributions of the diameter of VIP+ IN dendrites targeted by VGluT1+ or VGluT2+ axon terminals. Scale bars: ***A***, 250 μm; ***B***, 20 μm; ***C***, ***D***, 250 nm.

To confirm that VGluT1+ and VGluT2+ axon terminals indeed form synapses with VIP+ INs and to evaluate quantitatively postsynaptic target preference, we performed double immunopre-embedding EM experiments. To reveal the full dendritic domain of VIP+ INs, we injected into the BLA of VIP-IRES-cre mice a viral vector to express the reporter protein GFP in a cre-dependent fashion (Fig. 11*A*,*B*). The presence of VGluT1 (Fig. 11*C*) or VGluT2 was visualized by silver-intensified immunogold and the GFP content by immunoperoxidase reactions, respectively (Fig. 11*D*). In agreement with the light microscopy results, both VGluT1+ and VGluT2+ axon terminals formed synapses preferentially with dendritic shafts, but also formed sporadic synapses on both somata and spines (Figs. 1*D*,*E*, 10*E*,*F*). The dendrites targeted by VGluT1+ and VGluT2+ boutons showed a similar distribution (*p* = 0.08, two-sample Kolmogorov–Smirnov test) and range in terms of diameter (*p* = 0.08, Mann–Whitney test; Fig. 11*E*,*F*), in agreement with our light microscopy observations.

## Discussion

**Figure 12.**
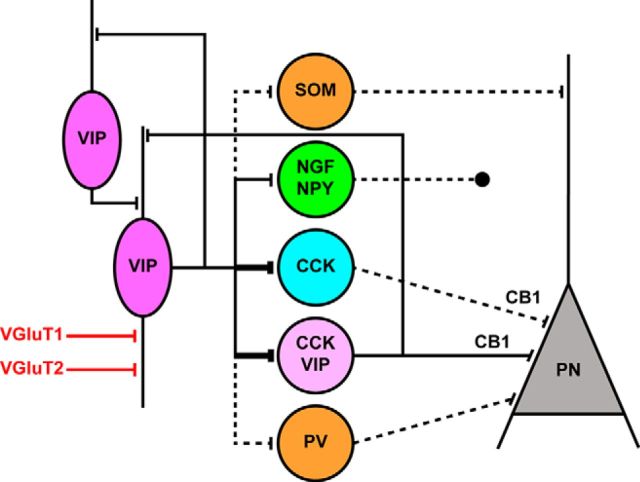
Schematic representation of VIP+ IN connectivity in the mouse BLA. Putative VIP+ IS-INs (magenta) primarily contact other IN subtypes: CCK+/CB1+ basket cells (cyan; comprising CCK+/CB1+/VIP+, light magenta), neurogliaform cells (NGF/NPY; green), and other IS-INs (VIP; magenta), in addition to SOM and PV (orange; Krabbe et al., 2016), which in turn innervate principal neurons (PN) at different plasma membrane domains. Therefore, VIP+ INs are in an ideal position to provide feedforward inhibition to these different INs and facilitate disinhibition of the entire somatodendritic domain of principal cells. An exception is represented by the neurogliaform cells (NGF/NPY) that do not form synapses (axon ending in a circle distant from the PN) onto principal neuron dendrites, but contribute to their inhibition via volume transmission (Mańko et al., 2012). CCK+/CB1+/VIP+ have reciprocal connections with VIP+ IS-INs. VIP+ IS-INs receive putative glutamatergic cortical (VGluT1) and subcortical (thalamic and hypothalamic) inputs (VGluT2). Black lines indicate GABAergic inputs and thicker black lines represent higher strength of innervation. Dashed black lines indicate connectivity reported in studies other than the present one. Red lines indicate glutamatergic inputs.

In this study, we provide the first comprehensive analysis of the distribution of VIP+ INs across the entire mouse BLA, as well as of their morphological and physiological properties. We also show that BLA VIP+ INs are heterogeneous. Based on different criteria, in agreement with the general consensus on IN classification (DeFelipe et al., 2013), we have identified VIP+ INs as belonging to IS-INs and CCK+ basket cells (Fig. 12). Through a combination of electrophysiology and optogenetics in different transgenic mouse lines, we revealed that VIP+ IS-INs innervate several classes of BLA INs, whereas CCK+/CB1+/VIP+ basket cells form, as expected, perisomatic synapses on principal neurons. Finally, as it is important to understand the inputs regulating the activity of VIP+ INs during relevant behavioral experiences, we show that VIP+ INs possess about the same number of VGluT1+ and VGluT2+ glutamatergic synapses on their dendrites. Furthermore, we found a relatively high density of GABAergic synapses on the somata and dendrites of VIP+ INs, most likely arising from other local BLA INs as well as from subcortical areas.

The occurrence of VIP+ INs in the BLA has been reported for a number of mammalian species (Lorén et al., 1979; Sims et al., 1980; Roberts et al., 1984; McDonald, 1985; Antonopoulos et al., 1987; Ni et al., 2014), including humans (Emson et al., 1979). This indicates that VIP+ INs in the BLA are well preserved throughout phylogenesis. None of these studies, however, examined either qualitatively or quantitatively the morphological and physiological features as well as connectivity of VIP+ INs. Our study demonstrates that the density of VIP+INs differs among the distinct subdivisions of the mouse BLA, suggesting a distinct impact of these INs on the local computation.

### Heterogeneity of BLA VIP+ INs

Our work reveals that BLA VIP+ INs largely follow the general organizational principles, although with some distinctive peculiarities, observed in other cortical areas (for review, see Kepecs and Fishell, 2014; Wang and Yang, 2018). In the mouse BLA, VIP+ INs are mostly bipolar or bitufted with small cell bodies and short axons projecting primarily near their somata. Combining anatomical, neurochemical, and electrophysiological methods with reporter mouse lines, we were able to show that BLA VIP+ INs consist of distinct subpopulations: CCK+/CB1+ basket cells and ≥2 types of IS-INs based on the CR content. The most prevalent IS-INs (50–60%) contained CR, although its density differed significantly among the different subnuclei, whereas the second was immunonegative for all the neuronal markers tested and was less frequent (30–40%).

CCK+/CB1+/VIP+ basket cells appeared more numerous in the LA (6–12%) than in the BA (4–5%), as further indicated by the higher proportion of VIP+ axon terminals colabeled with CB1 in the LA (∼18%) compared with the BA (∼3%). Previous reports from rats described a higher coexpression between VIP and CCK (Mascagni and McDonald, 2003). The reason for this discrepancy might be explained by methodological differences or analyzing techniques, although a species-specific difference cannot be ruled out. Functionally, we could also show that the active and passive membrane properties of VIP-containing basket cells were very similar to those of CCK+/CB1+ basket cells in the BLA (Barsy et al., 2017; Rovira-Esteban et al., 2017), whereas they significantly differed from those measured in VIP+ IS-INs. Combining *in vitro* recordings with optogenetics, we have then proven the ability of CCK+/CB1+/VIP+ basket cells to evoke IPSPs in ∼20% of principal neurons, as also confirmed by the suppression of the light-evoked IPSPs by the bath application of the CB1 agonist WIN 55,212-2. Only approximately two-thirds of BLA principal neuron somata were targeted by VIP+ boutons. We also observed that nearly half of the CB1+ varicosities apposed onto LA principal neurons were formed by CCK+/CB1+/VIP+ basket cells, whereas they were limited to only 15% onto BA principal neurons. This suggests that the contribution of CCK+/CB1+/VIP+ basket cells to the perisomatic inhibition of principal neurons varies significantly between the LA and BA nuclei. We found, moreover, that ∼20% of CCK+/CB1+/VIP+ axon terminals formed appositions onto other VIP+ INs, most likely IS-INs (Fig. 12).

In our study, most of the features of the recorded VIP+ IS-INs were comparable to analogous hippocampal and neocortical VIP+ INs (Tyan et al., 2014; Prönneke et al., 2015). As in other cortical regions (Acsády et al., 1996; Staiger et al., 1997, 2004) where VIP+ IS-INs preferentially, if not exclusively, innervate GABAergic cells, we also found that VIP+/CB1− boutons in the mouse BLA indeed target primarily GABAergic INs. Our work suggests that VIP+ IS-INs can be differentiated according to their spiking pattern into fast-spiking, low-spiking (or irregular-firing with spike broadening), and short-spiking (or accommodating), which on the other hand did not differ in passive and active membrane properties, morphological features, or coexpression of CR. Consistent with our findings, VIP+ INs with a high firing rate as well as with high initial spike frequencies followed by pronounced adaptation were previously observed in the mouse LA (Sosulina et al., 2010).

### VIP+ IS-INs innervate different classes of postsynaptic BLA INs

Genetic targeting and optical activation enabled us to demonstrate that VIP+ IS-INs give rise to GABA_A_ receptor-mediated postsynaptic responses in several BLA INs. Previous studies in the neocortex and hippocampus have consistently shown that VIP+ INs form synapses onto PV+ and SOM+ INs (Lee et al., 2013; Pfeffer et al., 2013; Pi et al., 2013; Sohn et al., 2016). Likewise, a recent report described how BLA VIP+ INs also innervate both PV+ and SOM+ INs (Krabbe et al., 2016). However, our approach did not accommodate tests for PV+ and SOM+ INs, as they do not express EGFP in the reporter line we used. On the other hand, we have identified additional classes of INs contacted by VIP+ IS-INs, which include CCK+ basket cells, neurogliaform cells, and other VIP/CR+ IS-INs. The number of postsynaptic INs within each of these different subtypes targeted by VIP+ IS-INs, and/or the strength of the innervation, appeared quite diverse, suggesting a complex network regulation, which requires further functional characterization.

Our work substantially extends previous findings and shows for the first time that VIP+ INs have a broad range of intrinsic postsynaptic targets, which include CCK basket and neurogliaform cells and IS-INs, in addition to PV+ and SOM+ INs. Particularly interesting is the innervation of neurogliaform cells as they evoke slow phasic inhibition of principal neurons by extrasynaptic release of GABA around their dendritic domains (Mańko et al., 2012).

### Synaptic inputs onto BLA VIP+ INs

The BLA receives extensive cortical and thalamic glutamatergic inputs (Sah et al., 2003). We demonstrate that VIP+ INs are direct targets of different excitatory afferents identified by their expression of VGluT1 and VGluT2. The synaptic inputs onto VIP+ INs identified by light microscopy were confirmed by EM. The dendritic shafts of VIP+ INs were the main subcellular domain targeted by glutamatergic inputs, with similar density on both the proximal and distal compartments. However, the density of excitatory inputs received by VIP+ INs appears much lower compared with other INs, such as those expressing PV (Smith et al., 1998; Gulyás et al., 1999; Andrási et al., 2017). One EM study of CR+ INs in the rat hippocampus revealed that they share a similar density of excitatory inputs to VIP+ INs (Gulyás et al., 1999), thus suggesting that the glutamatergic innervation of VIP+ and CR+ INs significantly differs at least from PV+ INs. Afferents to the BLA containing VGluT2 arise primarily from the thalamus and the ventromedial hypothalamus (Pitkänen, 2000; Fremeau et al., 2004). However, previous studies suggested that thalamic projections to the BA innervate dendritic spines of principal neurons almost exclusively (Carlsen and Heimer, 1988; LeDoux et al., 1991). Considering that a selective innervation of VIP+ INs by thalamic inputs would not be in conflict with a proportionally higher targeting of principal neuron spines, also in view of the relatively low density of these INs, tracing studies are needed to reveal the precise source of glutamatergic inputs containing VGluT2. The origin of VGluT1+ axon terminals, meanwhile, can arise from the prefrontal or other cortices as well as from the hippocampus, in addition to nearby principal neurons, since these are the primary areas projecting to the BLA and expressing VGluT1 (Pitkänen, 2000; Fremeau et al., 2004). In addition to excitatory inputs, VIP+ INs displayed a high density of GABAergic synapses, nearly twice that of other BLA INs (Smith et al., 1998; Klenowski et al., 2015). Indeed, most of the synaptic inputs to their somata were GABAergic, similar to VIP+ INs in the visual cortex (Hajós and Zilles, 1988). Our findings indicate that the interconnectivity among VIP+ INs accounts for only ∼10% of their GABAergic synapses, and suggest that the remaining 90% come from other BLA INs. In the rat BLA, Muller and colleagues (2003) reported synaptic connections between Calbindin+ INs and VIP+ INs. Previous reports in the neocortex have shown that VIP+ INs have strong reciprocal connections with PV+ (Staiger et al., 1997; Hioki et al., 2013; Sohn et al., 2016) and SOM+ INs (Lee et al., 2013; Pfeffer et al., 2013; Pi et al., 2013; Sohn et al., 2016). Future studies will have to determine whether VIP+ INs in the BLA also preferentially interconnect with PV+ and/or SOM+ INs.

### Functional considerations

Recent data suggest that VIP+ INs in cortical networks are critical for a functional disinhibition of principal cells (Kepecs and Fishell, 2014), as originally suggested by anatomical observations in the hippocampus (Acsády et al., 1996; Hajos et al., 1996). Disinhibition is emerging as a general mechanism for aversive learning that is not limited to the neocortex (Letzkus et al., 2015). Our findings suggest that VIP+ INs can be recruited by excitatory inputs, which relay sensory stimuli from both cortical and subcortical areas. The disinhibitory role of VIP+ INs may, therefore, provide a temporal window for firing of BLA principal neurons (Pouille and Scanziani, 2001; Wilent and Contreras, 2005) that may facilitate associative plasticity, e.g., the encoding of conditioned and unconditioned stimuli in fear conditioning. This requires a spatially and temporally coordinated regulation of the INs postsynaptic to VIP+ IS-INs and their cooperative activity to release principal neurons from inhibition. Our findings also indicate that a minority of VIP+ INs in the BLA are basket cells, whose specific role in circuit function remains to be elucidated. Future work using intersectional approaches combined with optogenetic manipulation of VIP+ INs in behaving animals will be an important next step in determining their role in BLA computation.
